# AKT2^S128^/CCTα^S315/319/323^-positive cancer-associated fibroblasts (CAFs) mediate focal adhesion kinase (FAK) inhibitors resistance via secreting phosphatidylcholines (PCs)

**DOI:** 10.1038/s41392-023-01728-6

**Published:** 2024-01-28

**Authors:** Jie Chen, Lingyuan Zhang, Yuheng Zhu, Di Zhao, Jing Zhang, Yanmeng Zhu, Jingyuan Pang, Yuanfan Xiao, Qingnan Wu, Yan Wang, Qimin Zhan

**Affiliations:** 1 Key Laboratory of Carcinogenesis and Translational Research (Ministry of Education/Beijing), Laboratory of Molecular Oncology, Peking University Cancer Hospital & Institute, 100142 Beijing, China; 2 Peking University International Cancer Institute, Peking University, 100191 Beijing, China; 3 Research Unit of Molecular Cancer Research, Chinese Academy of Medical Sciences, Beijing, China; 4 Soochow University Cancer Institute, Suzhou, 215000 China; 5 Institute of Cancer Research, Shenzhen Bay Laboratory, Shenzhen, 518107 China

**Keywords:** Gastrointestinal cancer, Molecular medicine

## Abstract

Abnormal metabolism is regarded as an oncogenic hallmark related to tumor progression and therapeutic resistance. Present study employed multi-omics, including phosphoproteomics, untargeted metabolomics and lipidomics, to demonstrate that the pAKT2 Ser^128^ and pCCTα Ser^315/319/323^-positive cancer-associated fibroblasts (CAFs) substantially release phosphatidylcholines (PCs), contributing to the resistance of focal adhesion kinase (FAK) inhibitors in esophageal squamous cell carcinoma (ESCC) treatment. Additionally, we observed extremely low levels of FAK Tyr^397^ expression in CAFs, potentially offering no available target for FAK inhibitors playing their anti-growth role in CAFs. Consequently, FAK inhibitor increased the intracellular concentration of Ca^2+^ in CAFs, promoting the formation of AKT2/CCTα complex, leading to phosphorylation of CCTα Ser^315/319/323^ sites and eventually enhancing stromal PC production. This activation could stimulate the intratumoral Janus kinase 2 (JAK2)/Signal transducer and activator of transcription 3 (STAT3) pathway, triggering resistance to FAK inhibition. Analysis of clinical samples demonstrated that stromal pAKT2 Ser^128^ and pCCTα Ser^315/319/323^ are related to the tumor malignancy and reduced patient survival. Pseudo-targeted lipidomics and further validation cohort quantitatively showed that plasma PCs enable to distinguish the malignant extent of ESCC patients. In conclusion, inhibition of stroma-derived PCs and related pathway could be possible therapeutic strategies for tumor therapy.

## Introduction

The prognosis for individuals suffering from esophageal squamous cell carcinoma (ESCC) is dismal, with the 5-year survival rate being less than 15%.^1–3^ Various risk factors, such as tobacco or alcohol addiction, genetic defects, and some other detrimental environmental factors may possibly induce the formation and development of ESCC. Importantly, the poor survival rate is resulted from the shortage for therapeutic efficacy from cytotoxic, targeted and immune-based therapeutics.^4,5^ Integrated multi-omics analysis of ESCC will yield precise molecular classification for exploring new diagnostics markers and therapeutic targets and then enhance the efficacy of ESCC treatment. Correspondingly, exploration of signaling addiction, vulnerability, or some other important tumor-related pathways and evaluation of their targetability and druggability can provide research paradigm for precision therapy against ESCC.

The critical mechanism of therapeutic resistances is the ESCC cells surrounding tumor microenvironment (TME), particularly its leading component-cancer-associated fibroblasts (CAFs).^6–8^ CAFs play the central role in the TME of solid tumors to induce various malignant phenotypes of tumors, including persistent growth, invasion and metastasis, angiogenesis, epithelial-mesenchymal transition (EMT), and the formation of tumor stem cells. Specifically, the crosstalk between ESCC and their surrounding CAFs makes vital impact on the biological behavior of tumor cells through cell-cell contact, cytokine release and exosomal transmission.^9–11^ Nevertheless, CAFs-derived metabolites, the vital signaling mediators, have ramifications for the biological role of tumor cells.^12–14^ The alteration of metabolites and their relevant intermediates effectively rewire tumor cells and the cellular components of TME to boost the output of lipid, protein, glucose and other important metabolism-related pathways. Furthermore, the expression changes or genetic mutations of key metabolic enzymes in tumor cells and their surrounding TME can dramatically elevate the concentration of metabolites in tumor cells and TME, and subsequently reshape TME and reprogram tumor cells to support the tumor malignancy and induce chemotherapy resistance. How tumor cells utilize metabolic nutrients and their affected signaling pathways are filed of concentrative investigation. With the purpose of managing the metabolic challenges imposed by the TME, tumor cells and CAFs cooperatively interact to facilitate tumor malignancy. Moreover, it remains unclear what are the metabolic profiles of CAFs and how CAFs-derived metabolites act on tumor malignancy and the response of tumor cells towards therapeutic agents.

Dysregulation of tumor-promoting kinases’ activities has been focused, due to tumor cells can utilize these proteins to enhance the tumor cells/TME interaction, and evade immune surveillance and then induce the malignant progression or metastasis of tumor cells. Selection of kinase targets and evaluation of their antitumor efficacy and related molecular mechanisms are critical for the development of antitumor agents. Focal adhesion kinase (FAK) refers to the cytoplasmic non-receptor protein tyrosine kinase and can be ubiquitously expressed.^15,16^ A lot of studies suggest that FAK overexpression in several types of solid tumors contributes to tumor malignancy and plays the role of the nexus to transit the TME-derived signaling into tumor cells.^16–20^ Under the stimulation of signalings from tumor cells themselves and cellular components of TME, intratumoral FAK can facilitate many cellular or biological activities or reactions of tumor cells via its kinase-dependent function, or act as scaffolding protein to influence the assembly of several protein signalosomes and resultantly promote the uncontrollable growth and sustained invasion and metastasis of tumor cells. These findings have contributed to developing FAK inhibitors for the clinical treatment of tumors. Many phase I or II clinical trials have been approved or conducted to observe the efficacy of FAK inhibitor alone or in combination with other antitumor agents in tumor treatment. Nevertheless, the clinical effect of FAK inhibitors remains controversial, even though some FAK inhibitors have made satisfying antitumor impact on preclinical studies only with in vitro assays.^15,21^ It can be hypothesized that this discrepancy is at least in part triggered by CAFs, secreting some substances to promote the dysregulation of intratumoral signaling pathways, as well as resultantly impairing the antitumor efficacy of chemotherapies.

While several studies have explored cytokines, chemokines or some growth factors secreted by tumor cells or CAFs mediate the crosstalk between these two types of cells. The in-depth understanding of TME-derived metabolites which regulate tumor and CAFs communications in therapeutic resistance of tumor cells still needs to be explored. In this work, we comprehensively exploited the ESCC CAFs-derived metabolic profiles and aimed at investigating whether CAFs-derived metabolites can be applied as biomarkers to identify the progression of tumor malignancy and how these metabolites change the antitumor effect of FAK inhibitors via the regulation of the intercellular signaling crosstalk between tumor cell and CAFs.

## Results

### CAFs impair the antitumor effect of FAK inhibitor in ESCC treatment

**Fig. 1:**
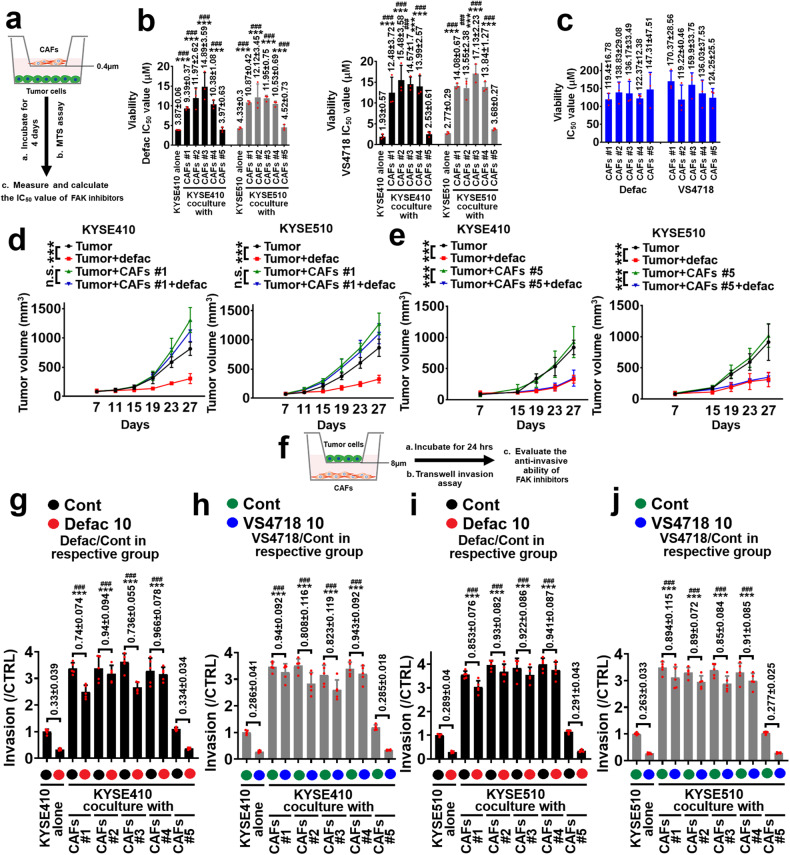
CAFs impairs the antitumor effect of defactinib. **a**, **b** Transwell apparatus with 0.4 μm pore size was used to evaluate the tumor growth inhibitory effect of defactinib and VS4718, the CAFs #1-#5 were respectively plated in the upper chamber of transwell plates. The KYSE410 or KYSE510 cells were respectively plated in the lower chamber of transwell plates (**a**). After cells were seeded, defactinib or VS4718 (0–10 μM) was added, incubated for 4 days, and then growth of indicated ESCC cells was measured using MTS assay. IC_50_ value of defactinib (left panel) or VS4718 (right panel) in KYSE410 and KYSE510 cells was shown. ***P < 0.001 as IC_50_ value of defactinib or VS4718 in KYSE410 and KYSE510 cells cultured alone compared with that of defactinib or VS4718 in KYSE410 and KYSE510 cells/CAFs #1 to #4 coculture system. ^###^*P* < 0.001 as IC_50_ value of defactinib or VS4718 in KYSE410 and KYSE510 cells/CAFs #1 to #4 coculture system compared with that of defactinib or VS4718 in KYSE410 and KYSE510 cells/CAFs #5 coculture system (**b**). **c** CAFs #1-#5 were treated with defactinib or VS4718 (0–25 μM) for 4 days, and the cell growth was evaluated using MTS assay. IC_50_ value of defactinib or VS4718 in CAFs was shown. Error bars, mean ± SD of three independent experiments. **d** KYSE410 (left panel) or KYSE510 (right panel) cells were respectively coinjected with CAFs #1 into the flank of BALB/c mouse. After the xenografts reached at approximately 80–100 mm^3^. Tumor cells with/without CAFs #1 were treated with control vehicle or defactinib (25 mg/kg/day, p.o.), respectively. Tumor volume was measured every 4 days for the indicated period. Curves of tumor volume were listed. **e** The experimental protocol of **e** was similar with that of **d** except the CAFs were chosen CAFs #5. n.s. no significant difference; ***P < 0.001. Error bars, mean ± SD of five independent experiments. **f** Transwell apparatus with 8 μm pore size was used to evaluate the anti-invasive ability of defactinib and VS4718, the CAFs #1-#5 were respectively plated in the lower chamber of transwell plates. The KYSE410 or KYSE510 cells were respectively plated in the upper chamber of transwell plates. After cells were seeded, 10 μM defactinib (**g**: in KYSE410 cells; **i**: in KYSE510 cells) or VS4718 (**h**: in KYSE410 cells; **j**: in KYSE510 cells) was added, incubated for 24 h, and then invasion of ESCC cells was measured using transwell assay. The invasive ratio of defactinib or VS4718/respective control was listed. ***P < 0.001 as the invasive ratio of defactinib or VS4718 in KYSE410 or KYSE510 cells cultured alone compared with that of defactinib or VS4718 in KYSE410 and KYSE510 cells/CAFs #1 to #4 coculture system. ^###^*P* < 0.001 as the invasive ratio of defactinib or VS4718 in KYSE410 and KYSE510 cells/CAFs #1 to #4 coculture system compared with that of defactinib or VS4718 in KYSE410 and KYSE510 cells/CAFs #5 coculture system

We first evaluated whether CAFs can affect the tumor inhibitory effect of FAK inhibitors, including defactinib and VS4718, using ESCC cell lines/CAFs (five cases of CAFs) coculture system in transwell apparatus with 0.4 μm pore size, and then tumor cells (in the lower chamber of transwell plates) were subjected to MTS assay (Fig. 1a). The IC_50_ values of defactinib or VS4718 (0–10 μM) in KYSE410 and KYSE510 cells cultured alone were 3.87 ± 0.06 and 4.33 ± 0.3 μM, or 1.93 ± 0.57 and 2.77 ± 0.29 μM (Fig. 1b and Supplementary Fig. 1a–d). In ESCC cells/CAFs #1–4 coculture system, the IC_50_ values of defactinib and VS4718 in KYSE410 or KYSE510 cells were higher than those of defactinib and VS4718 in KYSE410 or KYSE510 cells cultured alone (In KYSE410 cell/CAFs #1 to #4 coculture system, the IC_50_ values of defactinib (0–10 μM) were 9.39 ± 0.37, 11.97 ± 2.62, 14.89 ± 3.59, or 10.38 ± 1.08 μM; in KYSE510 cell/CAFs #1 to #4 coculture system, the IC_50_ values of defactinib (0–10 μM) were 10.87 ± 0.42, 12.12 ± 3.45, 11.95 ± 0.75, 10.53 ± 0.69 μM; in KYSE410 cell/CAFs #1 to #4 coculture system, the IC_50_ values of VS4718 (0–10 μM) were 12.48 ± 3.72, 15.48 ± 3.58, 14.57 ± 1.7, or 13.99 ± 2.57 μM; in KYSE510 cell/CAFs #1 to #4 coculture system, the IC_50_ values of VS4718 (0–10 μM) were 14.08 ± 0.67, 13.55 ± 2.38, 17.13 ± 2.23, 13.84 ± 1.27 μM) (Fig. 1b and Supplementary Fig. 1a–d). However, CAFs #5 could not increase the IC_50_ values of defactinib and VS4718 in ESCC cells treatment, compared with those of defactinib and VS4718 in KYSE410 or KYSE510 cell/CAFs #1 to #4 coculture system (In KYSE410 or KYSE510 cell/CAFs #5 coculture system, the IC_50_ values of defactinib (0–10 μM) were 3.97 ± 0.63, 4.52 ± 0.73 μM; in KYSE410 or KYSE510 cell/CAFs #5 coculture system, the IC_50_ values of VS4718 (0–10 μM) were 2.53 ± 0.61, 3.68 ± 0.27 μM) (Fig. 1b and Supplementary Fig. 1a–d). Furthermore, the IC_50_ values of defactinib and VS4718 (0–25 μM) in CAFs #1–5 were all greater than 100 μM, the exact IC_50_ values were indicated (Fig. 1c).

To assess whether CAFs contribute to the resistance of FAK inhibitor in ESCC treatment in vivo, KYSE410 or KYSE510 cells and CAFs #1 or #5 were respectively coinjected into BALB/c-nu mice. After tumor volume reached to approximately 100 mm^3^, animals were treated with defactinib. Defactinib decreased the tumor volumes of KYSE410 or KYSE510 tumor alone (The average tumor volume of KYSE410 or KYSE510 tumor alone at day 27 was 815.36 ± 113.94 or 865.67 ± 148.47 mm^3^; the average tumor volume of KYSE410 or KYSE510 tumor treated with defactinib at day 27 was 305.29 ± 86.64 or 328.35 ± 64.88 mm^3^), whereas could not hinder the tumor growth of these two ESCC tumors in the presence of CAFs #1 (The average tumor volume of KYSE410 or KYSE510 tumor in the presence of CAFs #1 at day 27 was 1308.65 ± 213.37 or 1272.19 ± 186.17 mm^3^; the average tumor volume of KYSE410 or KYSE510 tumor in the presence of CAFs #1 treated with defactinib at day 27 was 1115.25 ± 175.49 or 1106.21 ± 172.5 mm^3^) (Fig. 1d). CAFs #5 could not affect defactinib-mediated inhibition of tumor growth in indicated ESCC cells/CAFs #5 coinjection xenografted model (Fig. 1e).

To comprehensively evaluate the antitumor effect of defactinib in ESCC/CAFs coinjection xenografted model, the expression of Ki-67, CD31, and LYVE-1 in tumor tissues was measured with quantitative ELISA assays. As the results of Supplementary Fig. 2a–f shown, defactinib inhibited the expression of Ki-67, CD31 and LYVE-1 in KYSE410 and KYSE510 tumors (Supplementary Fig. 2a–c), but could not suppress the expression of these biomarkers in KYSE410 and KYSE510 tumors in the presence of CAFs #1 (Supplementary Fig. 2d–f). However, defactinib decreased the level of Ki-67, CD31 and LYVE-1 in KYSE410 or KYSE510 tumors/CAFs #5 (Supplementary Fig. 2g–i).

We then examined whether CAFs impaired FAK inhibitors-mediated inhibition of ESCC cells’ invasion using transwell apparatus with 8 μm pore size (Fig. 1f). Defactinib and VS4718 (10 μM) inhibited the invasion of KYSE410 and KYSE510 cells cultured alone (Fig. 1g–j). The invasive ratio of defactinib/control in KYSE410 or KYSE510 cells cultured alone was 0.33 ± 0.039 or 0.289 ± 0.04; the invasive ratio of VS4718/control in KYSE410 or KYSE510 cells cultured alone was 0.286 ± 0.041 or 0.263 ± 0.033. CAFs #1 to #4 enhanced the invasion ability of KYSE410 and KYSE510 cells in coculture system (Fig. 1g–j). Importantly, the invasive ratio of defactinib or VS4718/respective control in KYSE410 or KYSE510 cells/CAFs #1 to #4 coculture system was statistically higher than that of defactinib or VS4718 in KYSE410 or KYSE510 cells cultured alone. The invasive ratio of defactinib/respective control in KYSE410 cells/CAFs #1 to #4 coculture system was 0.74 ± 0.074, 0.94 ± 0.094, 0.736 ± 0.055, or 0.966 ± 0.078; in KYSE510 cells/ CAFs #1 to #4 coculture system was 0.853 ± 0.076, 0.93 ± 0.082, 0.922 ± 0.086, or 0.941 ± 0.087 (Fig. 1g, i). The invasive ratio of VS4718/respective control in KYSE410 cells/CAFs #1 to #4 coculture system was 0.94 ± 0.092, 0.808 ± 0.116, 0.823 ± 0.119, or 0.943 ± 0.092; in KYSE510 cells/ CAFs #1 to #4 coculture system was 0.894 ± 0.115, 0.89 ± 0.072, 0.85 ± 0.084, or 0.91 ± 0.085 (Fig. 1h, j). Furthermore, the invasive ratio of defactinib/respective control in KYSE410 or KYSE510 cells/CAFs #5 coculture system was 0.334 ± 0.034 or 0.291 ± 0.043 (Fig. 1g, i); the invasive ratio of VS4718/respective control in KYSE410 or KYSE510 cells/CAFs #5 coculture system is 0.285 ± 0.018 or 0.277 ± 0.025 (Fig. 1h, j). Invasive rates of FAK inhibitors in ESCC cells/CAFs #5 coculture system were significantly lower than those in KYSE410 or KYSE510 cells/CAFs #1 to #4 coculture system, indicating that CAFs #5 could not induce the anti-invasive resistance of FAK inhibitors in ESCC treatment.

The popliteal lymphatic metastasis model has been established by injecting cancer cells into the mice footpad and examining the draining popliteal lymph node, and the volume of lymph node reflects the degree of tumor cell metastasis.^12,22–24^ We have coinjected KYSE410 or KYSE510 cells/CAFs #1 or #5 into the footpads of mice, and then observed the anti-metastatic ability of defactinib in this xenografted model. As the results in Supplementary Fig. 3a shown, defactinib could not effectively inhibit the CAFs #1-facilitated formation of larger lymph nodes of KYSE410 and KYSE510 tumors. However, CAFs #5 could not affect the anti-metastatic ability of defactinib in the in vivo lymph node metastasis model (Supplementary Fig. 3b).

### Untargeted metabolomics and lipidomics identify that FAK inhibitor induces the secretion of phosphocholines (PCs) from CAFs

**Fig. 2:**
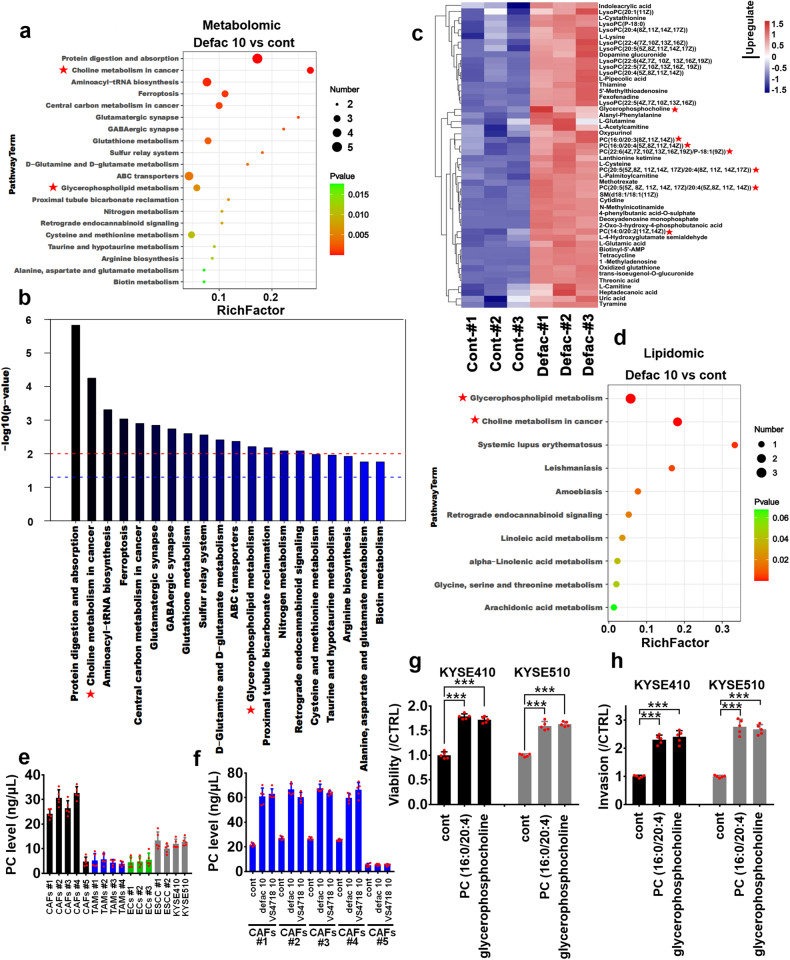
Defactinib stimulates PCs secretion from CAFs. **a**, **b** CAFs #1 was treated with control or 10 μM defactinib for 24 h, and then CM were collected, and subjected to untargeted metabolomics. The 20 enriched pathways have been shown using bubble chart (**a**) and bar chart (**b**). **c** The defactinib (10 μM) upregulated representative metabolites were shown using heatmap. **d** The experimental condition of **d** is consistent with **a**. The CM from CAFs #1 was subjected to lipidomics. The 10 enriched pathways were shown using bubble chart. Levels of PCs secreted from **e** 5 cases of CAFs, 4 cases of TAMs, 3 cases of ECs, 2 cases of ESCC and ESCC cell lines-KYSE410 and KYSE510 or **f** 5 cases of CAFs with/without 10 μM defactinib or VS4718, were evaluated using quantitative PCs ELISA assay. **g**, **h** KYSE410 and KYSE510 cells were treated with 10 μM PC (16:0/20:4) or glycerophospholipid for 4 days, then the growth of indicated ESCC cells was evaluated using MTS assay (**g**); or for 24 h, the invasion of indicated ESCC cells was evaluated using Transwell assay (**h**). ***P < 0.001. Error bars, mean ± SD of five independent experiments

Several studies have demonstrated that tumor microenvironment-derived metabolites can induce the resistance of tumor cells towards targeted agents.^25–27^ To explore how FAK inhibition might change the metabolic profile of CAFs, we performed an untargeted LC-MS-based metabolomic analysis in CAFs #1 treated with defactinib (10 μM). Several metabolic pathways, such as protein digestion and absorption (KEGG ID: hsa04974), choline metabolism in cancer (KEGG ID: hsa05231), central carbon metabolism in cancer (KEGG ID: hsa05230), glutathione metabolism (KEGG ID: hsa00480), ABC transporters (KEGG ID: hsa02010), or glycerophospholipid metabolism (KEGG ID: hsa00564), have been enriched upon defactinib treatment (Fig. 2a, b). Among these pathways, we have focused on choline and glycerophospholipid metabolisms, due to the upregulation of several glycerophospholipids, such as PC (16:0/20:4), PC (20:5/20:4), PC (14:0/20:2), PC (16:0/20:3), or glycerophosphocholine, in defactinib treatment (Fig. 2c).

We further investigated whether the secretion of choline-related metabolites from CAFs #1 could been stimulated by FAK inhibition using lipidomics. As shown in Fig. 2d and Supplementary Fig. 4a, b, glycerophospholipid (KEGG ID: hsa00564) and choline (KEGG ID: hsa05231) metabolisms have been enriched by defactinib (10 μM) treatment.

We evaluated the PCs secretion status of several cellular components of tumor microenvironment, including CAFs, tumor-associated macrophages (TAMs), endothelial cells (ECs), and primary ESCC cells, KYSE410, and KYSE510 cells. As shown in the results of Fig. 2e, the secreted concentrations of PCs from CAFs #1–4 were higher than those from CAFs #5, TAMs (4 cases), ECs (3 cases), primary ESCC cells (2 cases), KYSE410 and KYSE510 cells. Specifically, defactinib and VS4718 (10 μM) treatment stimulated the secretion of PCs from CAFs #1–4, whereas not from CAFs #5 (Fig. 2f). Furthermore, defactinib (10 μM) treatment inhibited the PCs secretion from KYSE410 and KYSE510 cells (Supplementary Fig. 5).

We chose two PCs-PC (16:0/20:4) and glycerophosphocholine for further functional assays to evaluate whether PCs induce the malignant progression of tumor cells, and found that these two PCs (10 μM) effectively stimulated the growth and invasion of KYSE410 and KYSE510 cells (Fig. 2g, h). CTP-phosphocholine cytidyltransferase (CCT) enzymes (including CCTα and β) catalyze the key rate-limiting step in choline pathway for phosphatidylcholine (PC) biosynthesis. We then knocked down CCTα and β in CAFs using siRNA, and further observed whether CCTα or β-depleted CAFs can contribute to the malignancy of ESCC cells. Depletion of CCTα in CAFs effectively blocked the secretion of PCs from CAFs (Supplementary Fig. 6a, b). Correspondingly, CCTα siRNAs impaired CAFs-induced the growth and invasion of KYSE410 and KYSE510 cells in ESCC cells/CAFs coculture system (Supplementary Fig. 6c, d). Furthermore, CCT inhibitor-miltefosine (25 μM) effectively inhibited the CAFs-induced ESCC malignancy (Supplementary Fig. 6e, f).

### CAFs-released PCs impair the antitumor effect of defactinib on ESCC cells

**Fig. 3:**
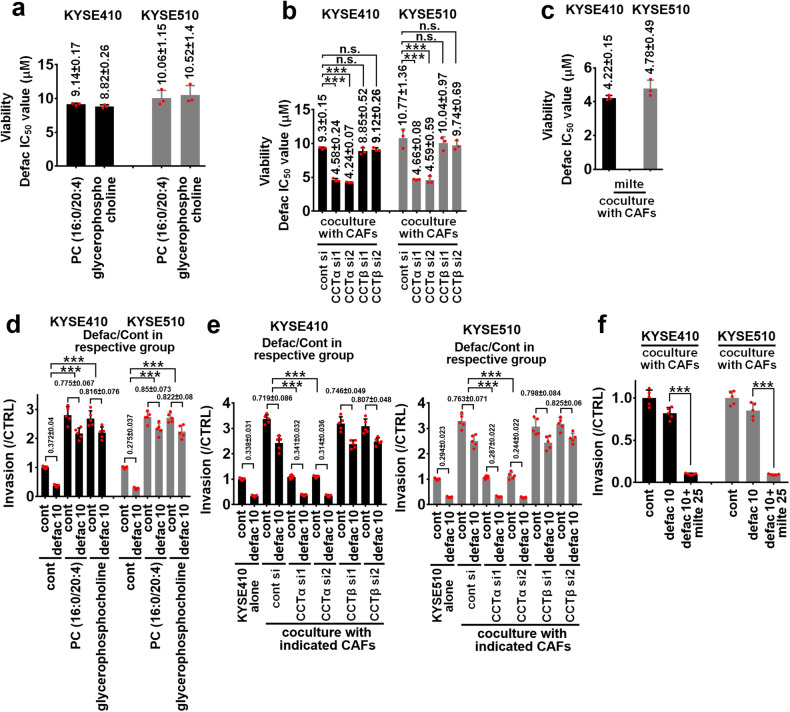
CAFs-released PCs induce the resistance of defactinib. **a** KYSE410 and KYSE510 cells were treated with 10 μM PC (16:0/20:4) or glycerophospholipid with defactinib (0–10 μM) for 4 days, then the growth of indicated ESCC cells was evaluated using MTS assay. IC_50_ value of defactinib in KYSE410 and KYSE510 cells was shown. **b** Transwell apparatus with 0.4 μm pore size was used to evaluate the CAFs-derived CCTα or CCTβ-mediated growth of tumor cells in the presence of defactinib. The control siRNA, CCTα siRNA1/2, or CCTβ siRNA1/2 CAFs #1 were plated in the upper chamber of transwell plates. The KYSE410 or KYSE510 cells were respectively plated in the lower chamber of transwell plates, and cocultured with indicated CAFs #1 with defactinib (0–10 μM) for 4 days, and then growth of indicated ESCC cells was measured using MTS assay. IC_50_ value of defactinib in KYSE410 and KYSE510 cells was shown. **c** CAFs #1 were plated in the upper chamber of transwell plates with 0.4 μm pore size. The KYSE410 or KYSE510 cells were respectively plated in the lower chamber of transwell plates, and cocultured with CAFs #1 with miltefosine (25 μM) and defactinib (0–10 μM) for 4 days, and then growth of indicated ESCC cells was measured using MTS assay. IC_50_ value of defactinib in KYSE410 and KYSE510 cells was shown. **d** KYSE410 and KYSE510 cells were treated with 10 μM PC (16:0/20:4) or glycerophospholipid in the presence of defactinib (10 μM) for 24 h, and the invasion of indicated ESCC cells was evaluated using Transwell invasion assay. **e** Indicated CAFs #1 were cultured in Transwell apparatus with 8 μm pore size, KYSE410 (left panel) or KYSE510 (right panel) cells were cultured in the upper chamber of transwell plates and treated with 10 μM defactinib for 24 h. The invasion of indicated ESCC cells was evaluated using Transwell invasion assay. **f** CAFs #1 was cultured in Transwell apparatus with 8 μm pore size, KYSE410 or KYSE510 cells were cultured in the upper chamber of transwell plates and treated with 10 μM defactinib with/without miltefosine (25 μM) for 24 h. The invasion of indicated ESCC cells was evaluated using Transwell invasion assay. The invasive ratio of defactinib/respective control was listed. n.s. no significant difference; ***P < 0.001. Error bars, mean ± SD of three to five independent experiments

We further evaluated CAFs-released PCs mediated the resistance of FAK inhibition in ESCC treatment. PC (16:0/20:4) and glycerophosphocholine (10 μM) induced the IC_50_ value of defactinib (0–10 μM) to 9.14 ± 0.17, or 8.82 ± 0.26 μM in KYSE410 cells, higher than that of defactinib in KYSE410 cells cultured alone (3.87 ± 0.06 μM) (Figs. 1b, 3a, and Supplementary Fig. 7a). The IC_50_ value of defactinib in KYSE410 cells/CAFs #1 CCTα siRNA1/2 coculture system was 4.58 ± 0.24, or 4.24 ± 0.07 μM, which was evidently lower than that in KYSE410 cells/CAFs #1 control siRNA coculture system (IC_50_ value of defactinib was 9.3 ± 0.15 μM) (Fig. 3b and Supplementary Fig. 8a). However, knockdown of CCTβ in CAFs #1 could not decrease the IC_50_ value of defactinib in coculture system (IC_50_ value of defactinib in CCTβ siRNA1 group was 8.85 ± 0.52 μM, and in CCTβ siRNA2 group was 9.12 ± 0.26 μM) (Fig. 3b and Supplementary Fig. 8a). CCT inhibitor-miltefosine (25 μM) effectively reduced the IC_50_ value of defactinib in KYSE410 cells/CAFs #1 coculture system to 4.22 ± 0.15 μM, lower than that in KYSE410 cells/CAFs #1 treated with defactinib alone (9.39 ± 0.37 μM) (Figs. 1b, 3c and Supplementary Fig. 9). Similar results were also obtained in KYSE510 cells (Figs. 1b, 3a–c, and Supplementary Figs. 7–9).

PC (16:0/20:4) and glycerophosphocholine (10 μM) blocked the anti-invasive effect of defactinib (10 μM) on KYSE410 and KYSE510 cells (Fig. 3d). CCTα siRNAs impaired CAFs #1-mediated the anti-invasive resistance of defactinib (10 μM) in ESCC treatment (Fig. 3e). Furthermore, miltefosine (25 μM) enhanced the anti-invasive ability of defactinib in ESCC cells in the presence of CAFs #1 (Fig. 3f).

### Phosphoproteomics identifies that the activation of AKT2/CCTα axis in CAFs contributes to the secretion of PCs

**Fig. 4:**
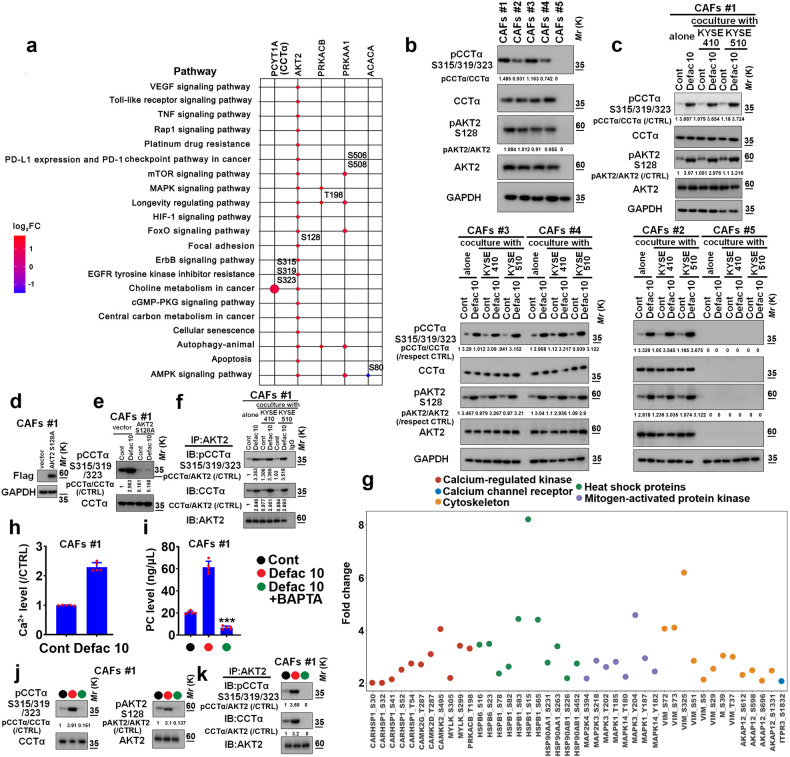
Defactinib stimulates the activity of AKT2/CCTα axis in CAFs. **a** CAFs #1 were treated with control or 10 μM defactinib for 24 h, and then cell lysates were collected, and subjected to phosphoproteomic analysis. The different KEGG pathways between control or defactinib (10 μM) were listed, and the expression pathways-related statuses of pCCTα Ser^315/319/323^ and pAKT2 Ser^128^ were shown. **b** Immunoblotting was used to measure the expression of pCCTα Ser^315/319/323^, CCTα, pAKT2 Ser^128^, or AKT2 in 5 cases of CAFs. GAPDH served as the internal control. **c** KYSE410 or KYSE510 cells were plated in the upper chamber of transwell plates with 0.4 μm pore size. The CAFs #1-#5 were plated in the lower chamber of transwell plates, and cocultured with/without 10 μM defactinib for 24 h. Then, lysates of CAFs #1-#5 were collected and subjected to immunoblotting assay for evaluating the expression of pCCTα Ser^315/319/323^, CCTα, pAKT2 Ser^128^, or AKT2. **d** CAFs #1 were stably transfected with control vector, loss-of-function AKT2 S128A plasmid, and the transfection efficacy was evaluated using immunoblotting to detect the expression of Flag. GAPDH was used as the loading control. **e** CAFs #1 harbored vector or loss-of-function AKT2 (S128A) plasmid were treated with/without defactinib (10 μM) for 24 h. The expression of pCCTα Ser^315/319/323^ and CCTα was evaluated using immunoblotting assay. **f** The experimental condition of (**f**) was similar with that of **c**. Then, lysates of CAFs #1 were immunoprecipitated with AKT2 (IP: AKT2). Immunocomplexes were subsequently immunoblotted using AKT2 (IB: AKT2), pCCTα Ser^315/319/323^ (IB: pCCTα Ser^315/319/323^) or CCTα (IB: CCTα) antibody. **g** Defactinib (10 μM)-upregulated the phosphorylation of several Ca^2+^-related proteins and their located pathways were shown. **h** CAFs #1 were treated with defactinib (10 μM), and the concentration of intracellular Ca^2+^ was quantified. **i**–**k** CAFs #1 were treated with defactinib (10 μM) in the presence or absence of Ca^2+^ chelator-BAPTA-AM (10 μM). The secreted PCs was evaluated using quantitative PCs ELISA assay (**i**). The expression of pCCTα Ser^315/319/323^, CCTα, pAKT2 Ser^128^, or AKT2 was measured using immunoblotting assay (**j**). The interaction between AKT2 and CCTα was evaluated using IP-IB assay. Lysates of CAFs #1 were immunoprecipitated with AKT2 (IP: AKT2). Immunocomplexes were subsequently immunoblotted using AKT2 (IB: AKT2), pCCTα Ser^315/319/323^ (IB: pCCTα Ser^315/319/323^) or CCTα (IB: CCTα) antibody (**k**). ***P < 0.001. Error bars, mean ± SD of five independent experiments

We further explored the molecular mechanism of the secretion of PCs from CAFs using phosphoproteomics. Interestingly, AKT2 Ser^128^ was appeared in almost all of these identified pathways in CAFs #1 treated with defactinib (10 μM), and the upregulated CCTα Ser^315/319/323^ sites existed in choline metabolism in cancer (KEGG ID: hsa05231) (Fig. 4a).

We found that AKT2 and CCTα and their phosphorylated forms-AKT2 Ser^128^ and CCTα Ser^315/319/323^ were highly expressed in CAFs #1–4 (AKT2^S128^/CCTα^S315/319/323^-positive CAFs), whereas not in CAFs #5 (AKT2^S128^/CCTα^S315/319/323^-negative CAFs) (Fig. 4b). We assessed whether FAK inhibition affected the phosphorylation of AKT2 Ser^128^ and CCTα Ser^315/319/323^ sites in CAFs using coculture system (transwell apparatus with 0.4 μm pore size). The upper chamber of transwell apparatus was plated with KYSE410 or KYSE510 cells, and the lower chamber was cultured with CAFs. After 24 h defactinib treatment, the lysates of CAFs were collected for evaluation of the phosphorylation status of AKT2 Ser^128^ and CCTα Ser^315/319/323^ sites. Defactinib (10 μM) effectively stimulated the phosphorylation of AKT2 Ser^128^ and CCTα Ser^315/319/323^ sites in CAFs #1–4 alone or in the presence of KYSE410 or KYSE510 cells (Fig. 4c). However, defactinib (10 μM) could not induce the expression of AKT2 and CCTα and their indicated phosphorylation status in CAFs #5 alone or cocultured with ESCC cells (Fig. 4c). Function-loss AKT2 (S128A) was stably transfected into CAFs #1, and then the effect of FAK inhibition on the phosphorylation of CCTα Ser^315/319/323^ was measured. Defactinib (10 μM) could not stimulate the phosphorylation of CCTα Ser^315/319/323^ sites in CAFs harbored AKT2 S128A mutant (Fig. 4d, e). Correspondingly, defactinib (10 μM) could not induce the secretion of PCs from CAFs #1 stably transfected with AKT2 S128A or CCTα S315/319/323A mutant (Fig. 4d, Supplementary Fig. 10a, b).

We examined the physical association between AKT2 and CCTα using immunoprecipitation assays, and found that defactinib (10 μM) facilitated the formation of AKT2/CCTα complex, and the phosphorylation of CCTα Ser^315/319/323^ in AKT2/CCTα complex in CAFs #1 alone or cocultured with indicated ESCC cells (Fig. 4f).

Interestingly, our phosphoproteomics data showed that defactinib (10 μM) could stimulate the phosphorylation of several Ca^2+^-related proteins in CAFs #1 (Fig. 4g). Correspondingly, we detected the level of intracellular Ca^2+^ upon defactinib treatment using calcium detection assay. Defactinib (10 μM) effectively induced intracellular Ca^2+^ levels in CAFs #1 (Fig. 4h). Importantly, CAFs #1 were pretreated with Ca^2+^ chelator-BAPTA-AM (10 μM), which effectively blocked defactinib-mediated the production of PCs, phosphorylation of AKT2 Ser^128^ and CCTα Ser^315/319/323^ sites, the formation of AKT2/CCTα complex and the activation of CCTα in this complex (Fig. 4i–k).

We then analyzed whether defectinib affects the phosphorylation of AKT2 and CCTα in ESCC cells. CAFs were cultured in the upper chamber of transwell apparatus (0.4 μm pore size), and the KYSE410 or KYSE510 cells were respectively cultured in the lower chamber of transwell apparatus. After 24 h defactinib treatment, the lysates of indicated ESCC cells were collected for assessing the phosphorylation status of AKT2 Ser^128^ and CCTα Ser^315/319/323^ sites. Defactinib (10 μM) inhibited the phosphorylation of AKT2 Ser^128^ and CCTα Ser^315/319/323^ sites in KYSE410 or KYSE510 cells with or without CAFs #1 (Supplementary Fig. 11).

### CAFs-derived AKT2/CCTα axis impairs the antitumor effect of defactinib on ESCC cells

**Fig. 5:**
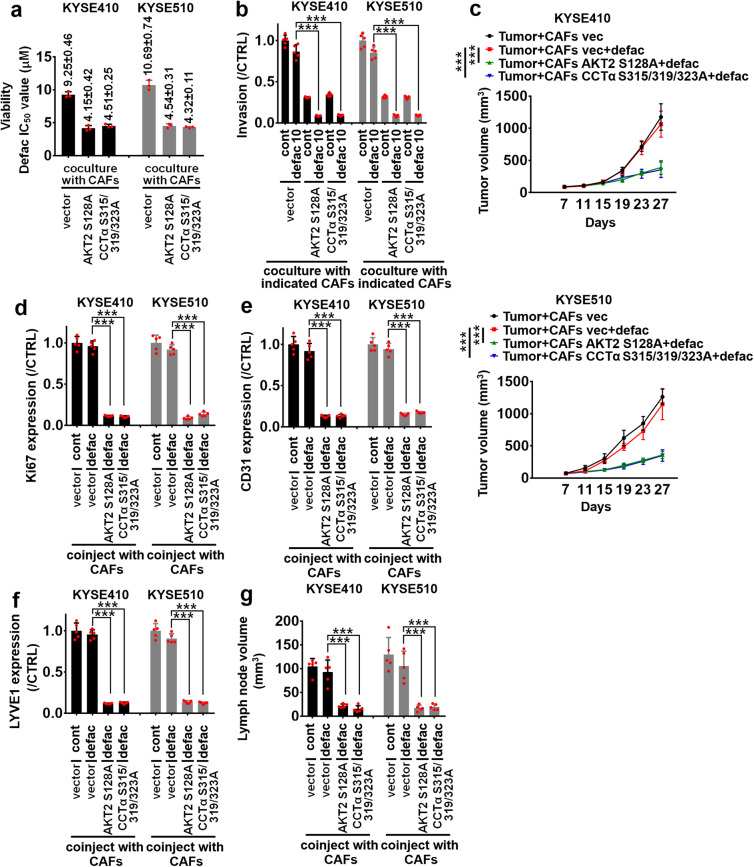
CAFs-derived AKT2/CCTα axis mediates the resistance of defactinib in ESCC treatment. **a** Transwell apparatus with 0.4 μm pore size was used to evaluate the growth inhibitory effect of defactinib. The CAFs #1 harbored vector, loss-of-function AKT2 (S128A) or CCTα (S315/319/323A) mutant were plated in the upper chamber of transwell plates. The KYSE410 or KYSE510 cells were respectively plated in the lower chamber of transwell plates. After cells were seeded, defactinib (0–10 μM) was added, incubated for 4 days, and then growth of indicated ESCC cells was measured using MTS assay. IC_50_ value of defactinib in KYSE410 and KYSE510 cells was shown. **b** Transwell apparatus with 8 μm pore size was used to evaluate the anti-invasive ability of defactinib (10 μM), the CAFs #1 harbored vector, loss-of-function AKT2 (S128A) or CCTα (S315/319/323A) plasmid were plated in the lower chamber of transwell plates. The KYSE410 or KYSE510 cells were respectively plated in the upper chamber of transwell plates. After cells were seeded, 10 μM defactinib was added, incubated for 24 h, and then tumor invasion was measured using transwell invasion assay. **c** KYSE410 (upper panel) or KYSE510 (lower panel) cells were respectively coinjected with CAFs #1 harbored vector, loss-of-function AKT2 (S128A) or CCTα (S315/319/323A) mutant into the flank of BALB/c mouse. After the xenografts reached at approximately 80–100 mm^3^. animals were treated with control vehicle or defactinib (25 mg/kg/day, p.o.), as indicated. Tumor volume was measured every 4 days for the indicated period. Curves of tumor volume were listed. After tumors were resected on day 27, the expression of Ki67 (**d**), CD31 (**e**) and LYVE1 (**f**) was assessed using quantitative ELISA assays. **g** A popliteal lymph node metastasis model was established in mice by inoculating the foot pads with KYSE410 or KYSE510 cells and CAFs #1 harbored vector, loss-of-function AKT2 (S128A) or CCTα (S315/319/323A) mutant. After 1 week, mice were treated with control vehicle or defactinib (25 mg/kg/day, p.o.) for 4 weeks. The lymph nodes were enucleated and lymph node volume was calculated. ***P < 0.001. Error bars, mean ± SD of three to five independent experiments

The IC_50_ value of defactinib (0–10 μM) in KYSE410 cells cocultured with CAFs #1 harbored with AKT2 S128A or CCTα S315/319/323 A mutant was 4.15 ± 0.42, or 4.51 ± 0.25 μM, respectively (Fig. 5a and Supplementary Fig. 12a). These IC_50_ values were lower than that of defactinib in KYSE410 cells/control vector CAFs #1 coculture system (IC_50_ value of defactinib was 9.25 ± 0.46 μM) (Fig. 5a and Supplementary Fig. 12a). Similar results were also obtained in KYSE510 cells (Fig. 5a and Supplementary Fig. 12b). Defactinib (10 μM) effectively inhibited the invasion of KYSE410 or KYSE510 cells in ESCC cells/CAFs #1 harbored AKT2 S128A or CCTα S315/319/323A mutant coculture system, compared with that of defactinib in KYSE410 or KYSE510/CAFs #1 harbored control vector coculture system (Fig. 5b). The results of in vivo assays, including subcutaneous co-transplantation of ESCC cells and CAFs #1 (Fig. 5c–f), or the popliteal lymph node metastasis model (Fig. 5g), confirmed those results obtained from in vitro assays. Taken together, these results indicated that CAFs-derived AKT2/CCTα axis critically contributes to the resistance of FAK inhibition in ESCC treatment.

### CAFs-released PCs activate intratumoral STAT3 to mediate the resistance of defactinib in ESCC treatment

**Fig. 6:**
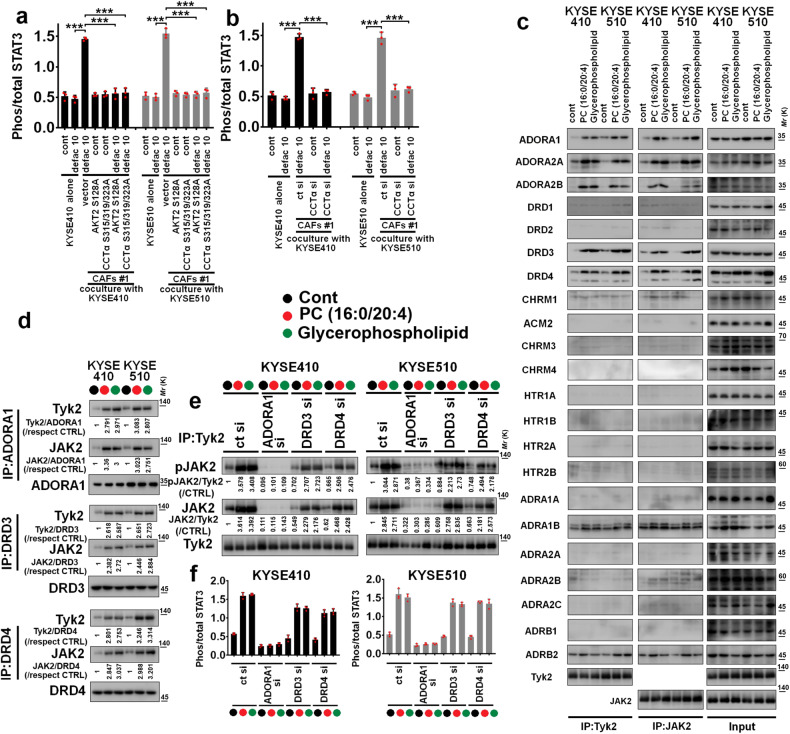
PCs mediate the formation of intratumoral ADORA1/Tyk2/JAK2 complex and then activate STAT3 to induce the resistance of FAK inhibitor in ESCC treatment. **a**, **b** Indicated CAFs #1 were plated in the upper chamber of Transwell apparatus with 0.4 μm pore size. The KYSE410 or KYSE510 cells were respectively plated in the lower chamber of transwell plates. After cells were seeded, 10 μM defactinib was incubated for 24 h, Then, lysates of KYSE410 and KYSE510 cells were collected, and the intratumoral STAT3 activity was evaluated using quantitative ELISA assay. **c** KYSE410 or KYSE510 cells were respectively treated with 10 μM PC (16:0/20:4) or glycerophospholipid for 24 h. Then, lysates of KYSE410 and KYSE510 cells were collected, incubated with Tyk2 antibody (left panel, IP: Tyk2), JAK2 antibody (middle panel, IP: JAK2) and input was also shown (right panel), and the expression of ADORA1, ADORA2A, ADORA2B, DRD1, DRD2, DRD3, DRD4, CHRM1, ACM2, CHRM3, CHRM4, HTR1A, HTR1B, HTR2A, HTR2B, ADRA1A, ADRA1B, ADRA2A, ADRA2B, ADRA2C, ADRB1, ADRB2, Tyk2, and JAK2 was shown. **d** The lysates from 10 μM PC (16:0/20:4) or glycerophospholipid-treated KYSE410 and KYSE510 cells were respectively immunoprecipitated with ADORA1, DRD3, or DRD4 antibody, and then subjected to immunoblotting with Tyk2, JAK2, ADORA1, DRD3, or DRD4. **e** KYSE410 or KYSE510 cells harbored control siRNA, ADORA1 siRNA, DRD3 siRNA or DRD4 siRNA were treated with control, 10 μM PC (16:0/20:4) or glycerophospholipid for 24 h. Then, lysates were immunoprecipitated with Tyk2 antibody, and subjected to immunoblotting with the expression of Tyk2, JAK2, or pJAK2 (**e**). **f** The experimental protocol of **f** was similar with that of **e**, and the lysates were subjected to STAT3 activity assay

Because intratumoral STAT3 contributed to the resistance of FAK inhibitor in tumor treatment.^28^ We evaluated whether defactinib stimulated the activation of intratumoral STAT3 in ESCC cells/CAFs coculture system, and found that defactinib (10 μM) upregulated the phosphorylation of STAT3 Tyr^705^ in KYSE410 and KYSE510 cells in the presence of CAFs #1, compared with defactinib in KYSE410 or KYSE510 cells cultured alone (Fig. 6a). We then determined whether PCs induce the activation of intratumoral STAT3, and found that PC (16:0/20:4) and glycerophosphocholine (10 μM) increased the phosphorylation of STAT3 Tyr^705^ in KYSE410 and KYSE510 cells (Supplementary Fig. 13a). Furthermore, AKT2 S128A, CCTα S315/319/323A mutant or CCTα siRNA effectively blocked CAFs (in the presence of defactinib)-induced intratumoral STAT3 activation in ESCC cells/CAFs #1 coculture system (Fig. 6a, b). Formation of Tyk2/JAK2 heterodimer is critical for persistent activation of intratumoral STAT3 and the resistance of targeted therapy, including ESCC cells.^24,29^ We evaluated whether PCs could stimulate the interaction between Tyk2 and JAK2 in ESCC cells, and found that PC (16:0/20:4) and glycerophosphocholine (10 μM) facilitated the formation of Tyk2/JAK2 complex and the activation of JAK2 in this complex (Supplementary Fig. 13b).

Because one member of G protein-coupled receptors (GPCRs)-platelet activating factor receptor (PAFR) interacted with Tyk2 and JAK2 to form protein complex and then persistently activate JAK2/STAT3 in ESCC cells,^24^ we hypothesized whether some members of GPCRs can participate into PCs-mediated assembly of Tyk2/JAK2 complex and the activation of JAK2/STAT3 pathway, and selected 22 GPCRs, including adenosine receptor A1 (ADORA1), ADORA2A, ADORA2B, dopamine receptor D1 (DRD1), DRD2, DRD3, et al for further co-IP assays. As the results shown in Fig. 6c, d, PC (16:0/20:4) and glycerophosphocholine (10 μM) increased interaction between ADORA1, DRD3, and DRD4 with Tyk2 and JAK2 in KYSE410 or KYSE510 cells, compared with KYSE410 or KYSE510 cells cultured alone (Fig. 6c, d). Then, the ADORA1, DRD3, and DRD4 were depleted using siRNAs, and ADORA1, DRD3, and DRD4-depleted KYSE410 and KYSE510 cells were treated with PC (16:0/20:4) and glycerophosphocholine (10 μM). As the results in Fig. 6e, f, and Supplementary Fig. 14a–c shown, ADORA1 depletion effectively disrupted the interaction between Tyk2 and JAK2, and inhibited the phosphorylation of JAK2 in Tyk2/JAK2 complex and the activation of STAT3 in indicated ESCC cells incubated with PC (16:0/20:4) or glycerophosphocholine.

Furthermore, coinhibition of FAK and JAK2/STAT3 pathways by defactinib (10 μM) combined with JAK2 inhibitors-ruxolitinib, or fedratinib (10 μM), or STAT3 inhibitor-S3I-201 (20 μM) effectively inhibited the growth and invasion of indicated ESCC cells in the presence of CAFs #1 (Supplementary Fig. 15a, b). The Results of in vivo xenografted models were similar with those of in vitro assays (Supplementary Fig. 15c–g).

### Stroma-derived AKT2/CCTα axis determines the ESCC progression and the survival of ESCC patients

**Fig. 7:**
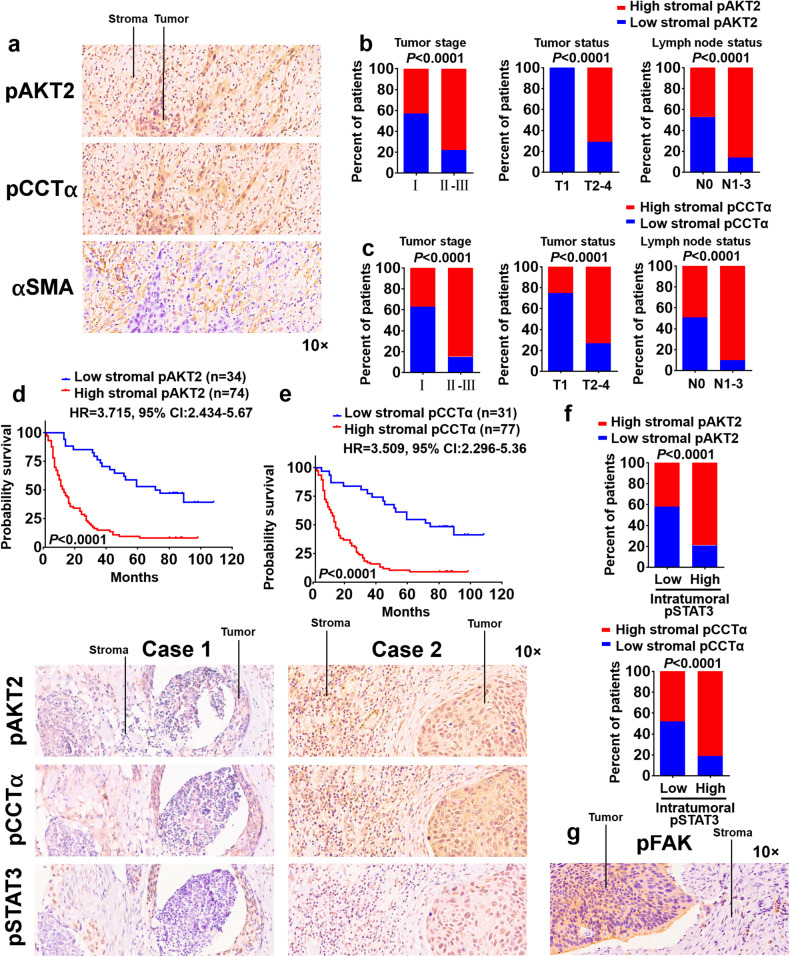
Stroma-derived AKT2/CCTα axis determines ESCC malignancy in clinical samples. **a** Representative images for immunohistochemical pAKT2 Ser^128^, pCCTα Ser^315/319/323^, or αSMA in 108 cases ESCC patients. Magnification, 10× as indicated. Percentages of 108 cases ESCC patients with high or low expression of stromal pAKT2 Ser^128^ (**b**) or pCCTα Ser^315/319/323^ (**c**) according to different clinical parameters as follows: tumor stage, tumor status and lymph node status. Two-tailed Pearson χ^2^ test. Kaplan–Meier curves of ESCC patients (108 cases) with low vs high expression of stromal pAKT2 Ser^128^ (**d**) or pCCTα Ser^315/319/323^ (**e**). **f** Stromal pAKT2 Ser^128^ or pCCTα Ser^315/319/323^ expression associated with intratumoral pStat3 Tyr^705^ expression in 108 cases ESCC specimens. Two representative specimens with low and high levels of stromal pAKT2 Ser^128^ or pCCTα Ser^315/319/323^ were shown. Magnification, 10× as indicated. Two-tailed Pearson χ^2^ test. **g** Representative images for immunohistochemical pFAK Tyr^397^ in ESCC patients. Magnification, 10× as indicated

We further determined the clinical expression of pAKT2 Ser^128^ and pCCTα Ser^315/319/323^ in ESCC stroma using immunohistochemistry (IHC) assay, and found that the expression of pAKT2 Ser^128^ (68.5%; 74/108) or pCCTα Ser^315/319/323^ (71.3%; 77/108) was high in tumor stroma (Fig. 7a). The expression of stromal pAKT2 Ser^128^ or pCCTα Ser^315/319/323^ was positively correlated with advanced-stage, higher-grade tumor status and lymph node status of ESCC tumors (Fig. 7b, c), and negatively correlated with the survival time of ESCC patients (Fig. 7d, e). Critically, stromal pAKT2 Ser^128^ and pCCTα Ser^315/319/323^ were coexpressed with the biomarker of CAFs-αSMA (Fig. 7a). The expression of CAFs-derived pAKT2 Ser^128^ or pCCTα Ser^315/319/323^ was positively correlated with the expression of intratumoral pSTAT3 Tyr^705^ (Fig. 7f).

Our previous study demonstrated that the expression of pFAK Tyr^397^ in ESCC tissues positively correlated with tumor malignancy.^30^ However, the staining intensity of pFAK Tyr^397^ was low in stroma (22.2% high expression of pFAK Tyr^397^; 24/108) (Fig. 7g).

### Prognostic performance of plasma concentrations of PCs for ESCC patients

To evaluate the correlation between concentrations of plasma lipids and ESCC malignancy, pseudo-targeted lipidomics was applied to quantitatively and comprehensively screen 1,000 lipids in plasma from 30 cases ESCC patients with 4 cases stage I and 26 cases stage II and III. 559 lipids were detected in the plasma of ESCC patients. Among these, plasma concentrations of some lipids were statistically higher in stage II and III group than stage I group (Supplementary Fig. 16a–n, and Supplementary Fig. 17a–q, and Supplementary Table 1). Importantly, several PCs, PC (18:1/22:1) (stage II and III group: median: 16.733 ng/mL, IQR: 14.0–21.9; stage I group: median: 12.414 ng/mL, IQR: 8.7–15.2), PC (18:1/20:5) (stage II and III group: median: 159.995 ng/mL, IQR: 122.1–263.9; stage I group: median: 87.203 ng/mL, IQR: 69.4–156.9), PC (18:1/20:1) (stage II and III group: median: 195.706 ng/mL, IQR: 173.1–237.1; stage I group: median: 146.432 ng/mL, IQR: 104.4–161.9), PC (18:0/22:1) (stage II and III group: median: 3.745 ng/mL, IQR: 3.3–5.4; stage I group: median: 2.816 ng/mL, IQR: 2.5–3.2), PC (18:0/20:1) (stage II and III group: median: 92.182 ng/mL, IQR: 72.6–112.2; stage I group: median: 66.871 ng/mL, IQR: 56.9–67.7), PC (18:0/18:1) (stage II and III group: median: 4424.936 ng/mL, IQR: 3774.9–5087.3; stage I group: median: 3552.909 ng/mL, IQR: 3172.6–3766.6), PC (16:0/22:1) (stage II and III group: median: 10.267 ng/mL, IQR: 8.4–12.5; stage I group: median: 7.392 ng/mL, IQR: 5.9–8.5), PC (16:0/20:1) (stage II and III group: median: 272.847 ng/mL, IQR: 222.5–292.9; stage I group: median: 204.171 ng/mL, IQR: 179.4–227.2), and metabolites of PCs-LPCs, including LPC (18:1) (stage II and III group: median: 3531.124 ng/mL, IQR: 2733.6–4025.1; stage I group: median: 2305.244 ng/mL, IQR: 2093.6–2943.7), LPC (20:1) (stage II and III group: median: 177.858 ng/mL, IQR: 138.8–211.0; stage I group: median: 115.585 ng/mL, IQR: 96.7–150.2), LPC (22:1) (stage II and III group: median: 16.167 ng/mL, IQR: 11.4-21.8; stage I group: median: 10.547 ng/mL, IQR: 9.1–12.9), LPC (22:2) (stage II and III group: median: 6.571 ng/mL, IQR: 5.7–7.8; stage I group: median: 5.101 ng/mL, IQR: 4.7–6.9), LPC (22:5) (stage II and III group: median: 1367.435 ng/mL, IQR: 1044.5–1668.4; stage I group: median: 922.245 ng/mL, IQR: 818.1–1042.9), LPC (24:1) (stage II and III group: median: 19.745 ng/mL, IQR: 16.2–23.4; stage I group: median: 12.639 ng/mL, IQR: 11.8–17.5), were statistically high in stage II and III group (*P* < 0.05, Mann–Whitney U test) (Supplementary Fig. 16a–n and Supplementary Table 1). Furthermore, other lipids, such as PS (18:0/22:5), PI (18:1/22:6), PE (18:1p/20:5), PE (18:1/22:4), PE (18:1/22:1), PE (18:0p/20:3), PE (18:0/20:1), PE (16:0p/20:5), PE (16:0p/20:3), LPE (24:1), LPE (22:1), LPE (20:1), LPE (19:0), LPA (20:0), FA (16:0), FA (22:6), or Hex2Cer (d18:2/24:1), were also statistically high in stage II and III group (Supplementary Fig. 17a–q and Supplementary Table 1). In the validation set, data from 89 ESCC patients showed that plasma PCs were positively correlated with the advanced-stage, higher-TN stages of ESCC patients (Supplementary Fig. 16o). Therefore, plasma PCs concentrations had a positive diagnostic performance and allowed evaluation of ESCC patients’ malignancy.

## Discussion

In the present study, we show that FAK inhibitors stimulate AKT2^S128^/CCTα^S315/319/323^-positive CAFs subset to secrete PCs, which induce malignant cells STAT3 activation to facilitate the therapeutic resistance of tumor cells. Present data establish a concept in CAFs-FAK-regulated and metabolites-mediated control of tumor malignancy with relevance to human ESCC with low stromal FAK expression, and detect potential novel actionable targets for anticancer therapy. Importantly, we found that plasma PCs can be served as biomarkers for classifying ESCC stage.

Our data show that FAK suppression increases the stromal level of PCs and their metabolites-LPCs, the major membrane structural phospholipids, and the stromal levels of other types of phospholipids, such as PE, PS, PI, or LPS. Moreover, FAK inhibition caused upregulation of unusual lipid subclass-the (*O*-acyl)-ω-hydroxy FAs (OAHFAs) from CAFs, suggesting that inhibiting FAK activity results in disruption of stromal choline and its related glycerophospholipid homeostasis, which could contribute to the resistance of FAK inhibition in tumor treatment. Furthermore, our data show that FAK inhibition increases CAFs-released ceramide (CER) and sphingomyelin (SM) levels, whose productions are induced by cellular stress response.^31,32^ It is possible that FAK inhibitors may function as an exogenous stress to dysregulate the choline homeostasis in stromal cells, due to the low expression of stromal FAK, which mediates no available target for FAK inhibitors exerting their anti-signaling function and subsequent anti-growth effect.^33^ Overall, dysregulated choline homeostasis and enhanced cellular stress work together to mediate FAK inhibition-induced secretion of PCs from CAFs to mediate the resistance of FAK inhibitor.

Our MS-based phosphoproteomics indicated that AKT2, the stress-induced protein kinase,^34,35^ was able to effectively stimulate the production of PCs from CAFs after FAK inhibitor treatment. AKT2 is an important signaling regulator of metabolism and can be stimulated to counteract stress-induced apoptosis.^36^ Previous study has indicated that stress-responsive FKBP51 activated AKT2 signaling to enhance glucose uptake in skeletal myotubes.^37^ We identified that FAK inhibition promoted the phosphorylation of the key rate-limiting step enzyme of PC biosynthesis-CCTα at Ser^315/319/323^ sites, and then triggered the overproduction of PCs from CAFs. Clearly, the control of CCTs activity is complex and that is involves multiple oncogenic signaling pathways-related factors that modulate expression and function of CCTs.^38,39^ Using a combination of phosphoproteomics and functional assays, we further discovered that AKT2 interacted with CCTα and induced the phosphorylation of CCTα to improve its activity in stromal cells. When stroma-derived metabolites harshly elevated, tumor cells could quickly utilize these metabolites to boost their own growth and resistance to the cytotoxic effect of chemotherapies.^38,40^ Importantly, therapeutic strategies by unselectively targeting whole CAFs population are ineffective since the existence of CAFs heterogeneity. We found that CAFs#1 to #4 (AKT2^S128^/CCTα^S315/319/323^-positive CAFs) produced the similar effect to induce the ESCC malignancy and impair the antitumor effect of FAK inhibitors in in vitro assays. Thus, we randomly chosen CAFs#1 for further xenograft model and omics assays; in subsequent assays, we have further validated the change of PCs in other CAFs, and confirmed that CAFs #2 to #4 can produce similar biological effects to CAFs #1. ESCC cells have not responded to coculture with CAFs#5 (AKT2^S128^/CCTα^S315/319/323^-negative CAFs). We also found that AKT2^S128^/CCTα^S315/319/323^-positive CAFs subset provides the adequate supply of PCs for the persistent activation of intratumoral STAT3 maintained by the Tyk2/JAK2 complex, and resultantly induced FAK-targeted therapy resistance and the ESCC malignancy. Thus, targeting stromal AKT2/CCTα axis and their-derived PCs has been suggested as an effective strategy for enhancing the antitumor effect of FAK inhibitor. Specifically, our data also indicate that FAK inhibition-stimulated the activation of AKT2/CCTα axis and PC production uniquely occurred in CAFs, while not in tumor cells. Combination with our previous report that FAK inhibition could effectively inhibit the expression of several metabolism-related molecules and the malignancy of ESCC cells cultured alone. We suggested that identifying the metabolic processes operating in specific CAFs subsets could provide an opportunity for developing novel antitumor strategies.

Hostile microenvironmental conditions within tumors, including nutrient deprivation, oxygen limitation, high metabolic demand, oxidative stress, and drug stimulation, provoke persistent stress to endow malignant cells with greater tumorigenic, metastatic, and drug-resistant capacity.^41^ Specifically, calcium signaling pathways have been identified to exert important roles in the establishment and maintenance of drug resistance.^42,43^ Combined these findings with our data, we suggest that rapidly rising Ca^2+^ concentration in stroma and increasing the production of stromal metabolites conferred increased resistance to cell death from stress or apoptotic stimuli, providing a drug-resistant stromal niche for TME-mediated tumor malignancy. Thus, the balance between signaling alterations caused by direct effects of FAK inhibition in cancer cells and stromal fibroblasts could potentially be vital to determine the overall treatment outcome.

Drug resistance is the commonly observed issue when targeted therapy is deployed in both preclinical and clinical settings. Constitutive activation of STAT3 has frequently been found in several types of solid tumors, and mediates resistance to conventional chemotherapy and targeted therapy.^44–46^ In current work, we have uncovered a TME-derived metabolites-induced STAT3 activation in response to FAK inhibition. We offer novel evidence that the crosstalk between TME and tumors is critical for driving the targeted therapy response. Correspondingly, ruxolitinib, baricitinib and S3I-201, targeting different levels of the STAT3 signaling cascade, showed a strong synergism with FAK inhibitor both in vitro and in vivo. Importantly, we have found that FAK inhibitors are unable to exert inhibitory effect on the growth of CAFs, but can facilitate the secretion of PCs from CAFs. Although STAT3 is the important signaling protein in CAFs, and activated STAT3 effectively induces the tumor-promoting function of CAFs, the aim of present study is not to evaluate the tumor-promoting effect of JAKs/STAT3 pathway in CAFs.^24,47,48^ We have focused on the inhibition of intratumoral FAK and JAK2/STAT3 pathways on the malignant progression of ESCC cells in the presence of CAFs-derived PCs. We will explore the effect of JAKs/STAT3 signaling pathway on the secretion of PCs or even other lipid metabolites from CAFs.

Present study highlights a previously unclear role of high plasma PCs in facilitating tumor progression and may be exploited as targets for therapeutic development against solid tumors. Accumulating reports have indicated the relationship between metabolites and the development of tumors.^49–51^ In light of our findings, we speculated that high concentration of plasma PCs in ESCC patients play a critical role in ESCC malignancy. Inhibition the effect of PCs on tumor cells can effectively block tumor malignant progression. Taken all together, plasma PCs levels can not only be used as biomarker to discriminate tumor stages but also be utilized as a potential target for tumor treatment or enhancement the antitumor efficacy of targeted therapies.

In conclusion, combining multi-omics, we systematically investigated PCs-based paracrine communication between specific subset of CAFs and tumor cells to limit the antitumor efficacy of FAK inhibitors. Mechanistically, the alteration of CAFs-derived AKT2/CCTα axis and its-activated intratumoral JAK2/STAT3 pathway induces the resistance of FAK inhibitor in tumor treatment. Importantly, PCs can potentially be used as new biomarkers for ESCC diagnosis. These data provide a new strategy for targeting metabolites-related pathway for ESCC treatment (Supplementary Fig. 18).

## Methods and materials

### Antibodies and reagents

All information of antibodies and reagents were listed in Supplementary Table 2.

### Cell culture and transfection

ESCC cell lines-KYSE410 and KYSE510 were provided by Dr. Yutaka Shimada (Kyoto University). The primary CAFs, ESCC cells, TAMs, or ECs were isolated from fresh ESCC tissues (clinical stage: II) using magnetic-activated cell sorting (MASC) with anti-FSP (fibroblast specific protein, Miltenyi Biotec, Cat # 130-050-601), anti-CD326 (EpCAM, Miltenyi Biotec, Cat # 130-061-101), anti-CD14 (Miltenyi Biotec, Cat # 130-050-201), or anti-CD31 (Miltenyi Biotec, Cat # 130-091-935) microbeads according to manufacturer’s instructions. All cells were cultured in RPMI-1640 medium contained with 10% heat-inactivated FBS (Gibco) and 1% penicillin/streptomycin in a 37 °C humidified incubator under 5% CO_2_.

The siRNA-based approach was applied to generate targeted genes-knockdown cells. Indicated siRNAs were transfected into primary CAFs using Lipofectamine 2000 reagent. For plasmid stable transfection, pcDNA 3.1-Flag plasmid contained AKT2 S128A or CCTα S315/319/323A mutant was transfected into CAFs. Subsequently, positive clones were selected for further experiments. Tansfection efficacy was evaluated using immunoblotting. Sequences of siRNAs were listed in Supplementary Table 3.

### Immunoprecipitation (IP) and immunoblotting (IB) analysis

For IP assay, indicated cells were washed with PBS, lysed in NP40 buffer supplemented with protease and phosphatase inhibitors for 30 min, and then centrifuged at 12000 *g* for 20 min at 4 °C. Supernatants were collected to incubated with indicated primary antibodies (approximately 10 μg antibody/sample) and protein A/G sepharose beads (ThermoFisher) on a rotator at 4 °C overnight. Then, samples were centrifuged at 4 °C for 5 min at 3000 g, supernatants were discarded, and pellets were washed with 800 μL cold NP40 buffer for 3 times. Finally, beads were collected, and 60 μL loading buffer was added to the beads. The beads were bathed in metal for 5 min, and supernatants were subjected to IB assay.

For IB assay, proteins were separated using sodium dodecyl sulfate (SDS)-PAGE, and transferred onto a nitrocellulose (NC) membrane. After blocking with PBS buffer solution containing 0.1% Tween-20 and 5% nonfat milk for 1 hour, the membranes were incubated with indicated primary antibodies at 4 °C overnight. PBST was used to wash NC membranes for three times. The membranes were incubated with secondary antibodies for 1 h, and then washed an additional three times with PBST and detected by chemiluminescence (ThermoFisher).

### Xenograft study

Female BALB/c-nu mice (purchased from Beijing Vital River Laboratory) with 3, 4 weeks of age were used in present assay. All animal procedures were approved by Institutional Review Board of Peking University Cancer Hospital & Institute.

KYSE410 or KYSE510 cells were subcutaneously co-injected with indicated CAFs into the flank of mice.^24,52,53^ When the tumor reached around 100 mm^3^, defactinib (25 mg /kg/day, p.o) alone or in the presence of ruxolitinib (10 mg/kg/day, p.o.), or fedratinib (10 mg/kg/day, p.o.), or S3I-201 (25 mg/kg/day, p.o.) for consecutive 3 weeks (*n* = 5/group). Tumor volume was evaluated using our reported formula.^12,24^ Human Ki67, CD31, or LYVE-1 ELISA kits (Raybiotech) were applied to measure the proliferation, angiogenesis, or lymph-angiogenesis of indicated ESCC tumors.^24^ The experimental protocols were according to manufacturer’s instructions.

For evaluation of lymph node metastasis of ESCC cells, KYSE410 or KYSE510 cells were subcutaneously co-injected with indicated CAFs into the footpads of mice (*n* = 5/group). The agents used in this assay was consistent with the model that subcutaneous tumor cells inoculation. Treatment was started from week 2 and sustained for 4 weeks. Lymph node volume was evaluated by our reported formula.^12,24^

### ESCC tissues and IHC staining

All procedures and experiments of ESCC tissues were approved by the institutional Review Board of Peking University Cancer Hospital. The protocols of IHC staining and the calculation of staining index were according to our previous studies.^12,30^ The dilution of primary antibodies was as follow: pFAK Tyr^397^ (1:100), pAKT2 Ser^128^ (1:100), pCCTα Ser^315/319/323^ (1:500), pSTAT3 Tyr^705^ (1:4000), or αSMA (1:1500).

### Statistical analysis

All data are expressed as the mean ± SD, and statistical analyses are performed by Graphpad software. Unpaired Student’s *t* test (two-tailed) was applied to compare the difference between two groups.^54–59^ For analysis of clinical IHC samples, Chi-square test was used to evaluate the correlation between two factors. Kaplan–Meier method was employed to establish the survival curves of ESCC patients. *P*-value < 0.05 was considered statistically significant.

Other methods and materials were included in Supplementary file.

### Supplementary information

Supplementary Figures and Figure legends

Supplementary Table 1

Supplementary Tables 2 and 3

## Data Availability

The phosphoproteomic data have been deposited in https://www.iprox.cn/page/home.html, and the accession number was: PXD032254. All data in present article are available upon reasonable request from the corresponding authors.
