# Supplementary material for: AKT2S128/CCTαS315/319/323-positive cancer-associated fibroblasts (CAFs) mediate focal adhesion kinase (FAK) inhibitors resistance via secreting phosphatidylcholines (PCs)
Source: Signal Transduct Target Ther. 2024 Jan 28;9:21. doi: 10.1038/s41392-023-01728-6 (PMC10821909; doi:10.1038/s41392-023-01728-6)
Supplement: Supplementary file 1 — Supplementary Figures and Figure legends [file 41392_2023_1728_MOESM1_ESM.doc]

Supplementary Materials for

**AKT2^S128^/CCTα^S315/319/323^-positive cancer-associated fibroblasts (CAFs) mediate focal adhesion kinase (FAK) inhibitors resistance via secreting phosphatidylcholines (PCs)**

Jie Chen^1,2,3,4#^*, Lingyuan Zhang^1,2,3#^, Yuheng Zhu^1#^, Di Zhao^1,2,3^, Jing Zhang ^1,2,3^, Yanmeng Zhu^1,2,3^, Jingyuan Pang^1^, Yuanfan Xiao^1,2,3^, Qingnan Wu^1,2,3^, Yan Wang^1,2,3^ and Qimin Zhan^1,2,3,4,5^*

Correspondence to: [zhanqimin@bjmu.edu.cn](mailto:zhanqimin@bjmu.edu.cn), cj_blue@126.com

**This file includes:**

Materials and Methods

Figures.S1 to S18

**Supplementary Materials and Methods**

**Proliferation assay**

For evaluation of CAFs-mediated FAK inhibitor resistance in ESCC treatment, CAFs were plated in the upper chamber of transwell apparatus (0.4 μm insert), and ESCC cells were cultured in the lower chamber. Defactinib or VS4718 (0-10 μM) or/and other inhibitors, including miltefosine (25 μM), ruxolitinib, fedratinib (10 μM), or S3I-201 (20 μM), were treated for 4 days, and then the upper chamber was discarded. The growth rate of ESCC cells was assessed using MTS assay (Progema). The IC_50_ value of defactinib and VS4718 was listed in presented Figures.

**Invasion assay**

EZCell^TM^ cell invasion assay kit (Biovision) was applied to evaluate CAFs-mediated defactinib resistance in ESCC cells treatment. Briefly, indicated ESCC cells were plated in the upper chamber of transwell apparatus (8 μm insert), and indicated CAFs, PC (16:0/20:4) and glycerophosphocholine (10 μM) were added to the lower chamber. Defactinib or VS4718 (10 μM) or/and other inhibitors, including miltefosine (25 μM), ruxolitinib, fedratinib (10 μM), or S3I-201 (20 μM), were treated for 24 hours, and then remove the non-invasive cells from the top chamber using a cotton swab. The invasive cells were obtained and incubated with dissociation solutions and cell dye for 1 hour at 37 °C in CO_2_ incubator.

**Concentration of phosphatidylcholine (PC) in condition media (CM) and plasma of ESCC patients**

PC assay kit (Sigma-Aldrich) was used to measure the levels of PC in the CM from indicated ESCC cells and CAFs, or ESCC patients’ plasma, which included plasma of 89 cases ESCC patients (stage Ⅰ: 20 cases, stage Ⅱ-Ⅳ: 69 cases; T1: 21 cases, T2-4: 68 cases; N0: 32 cases, N1-4: 57 cases). Supernatants of indicated cells were collected, and centrifuged at 3000 rpm for 10 minutes to obtain CM. Then, 50 μL sample buffer (44 μL CM, 2 μL PC hydrolysis enzyme, 2 μL PC development mix, and 2 μL fluorescent peroxidase substrate) was combined with 50 μL reaction mix, and incubated at room temperature for 30 minutes. Then, OD value was measured spectrophotometrically at 570 nm. The ESCC patients’ plasma was directly subjected to PCs assay.

**Intracellular concentration of Ca^2+^**

CAFs were collected and suspended in 500 μL calcium assay buffer and put on ice for quickly pipetting. Then, intracellular concentration of Ca^2+^ was evaluated using Ca^2+^ detection kit (Nanjing Jiancheng Bioengineering Institute). Briefly, 50 μL sample were incubated with 500 μL MTB reagent, 1 mL alkaline solution, and 50 μL protein clarifier for 5 minutes. OD value was measured spectrophotometrically at 610 nm.

**STAT3 activity**

Human phospho-STAT3 Tyr^705^ and total STAT3 ELISA kit (Raybiotech) was used to evaluate the activity of intratumoral STAT3. The protocols were according to manufacturer’s instruction and our previous study.^1^

**Phosphoproteomic analysis**

**Protein extraction and tryptic digestion**

CAFs samples were washed with three times with phosphate buffer saline (PBS), and then lysed in SDS lysis buffer supplemented with PMSF and phosphatase inhibitors (Cat # 4906837001, Roche). Samples were sonicated with 3 min (1 s on and 1 s off, 80 W power) on ice and centrifuged at 12, 000 *g* for 10 min to remove insoluble debris. The supernatants were collected, and the protein concentration was measured using BCA protein assay (Cat # 23225, ThermoFisher). Extracted proteins were reduced in 5 mM DTT at 55 °C for 30 min and then alkylated in 10 mM iodoacetamide at room temperature for 15 min in darkness. Six times of the volume of precooled acetone was added to above system to precipitate the proteins, placed samples at -20 °C overnight, and then centrifuged at 8, 000 *g* for 10 min to collect the precipitate. Samples underwent trypsin digestion (enzyme-to-substrate ratio of 1:50 at 37 °C overnight) and lyophilized.

**The enrichment of phosphorylated peptides**

For iTRAQ labeling, the lyophilized samples were resuspended in 60 μL 100 mM TEAB solution and then transferred into 1.5 mL Eppendorf tube for labeling. 70 μL isopropanol was added to iTRAQ reagent vial (Cat # 4381663, ABSCIEX) at room temperature, and then vortexed for mixing, centrifuged. This step was repeated 1 time. 100 μL iTRAQ label reagent was added to samples for mixing. Tubes were incubated at room temperature for 2 h. Finally, 200 μL HPLC water was added to each sample and incubated for 30 min to stop reaction. The labeled peptides samples were lyophilized and stored at -80 °C.

For phosphopeptides enrichment, the high-select^TM^ Fe-NTA phosphopeptide enrichment kit was used (Cat # A32992, ThermoFisher). Briefly, the labeling peptides were incubated with 200 μL binding/washing buffer in spin columns and incubated for 30 min. Then, columns were placed into microcentrifuge tubes for centrifuging at 1, 000 *g* for 30 s, and the flowthrough was discarded. Bound columns were washed by 200 μL binding/washing buffer, and then centrifuged at 1000 *g* for 30 s. This step was repeated for three times. Bound peptides were washed with 200 μL HPLC water to remove the unphosphorylated peptides. The remaining peptides were added with 100 μL elution buffer, resolved on columns, and then centrifuged at 1, 000 *g* for 30 s. The incubation reaction was repeated. The final enriched phosphopeptides were immediately vacuum concentrated for further use.

**LC-MS/MS**

Peptides samples were analyzed on an EASY-nLC 1200 system (ThermoFisher) coupled with a TimsTOF pro mass spectrometry (Bruker). Peptides were re-dissolved in mobile phase A (H_2_O-FA; 99.9: 0.1, v/v) and immediately loaded onto the 25 cm long, C18 analytical column (75 μm inner diameter; Cat #AUR2-25075C18A; Ionopticks).

Peptides were separated at a fluent flow rate of 400 nL/min. Peptide separation was achieved with a 90 min gradient (0-66 min, 2 to 21% of buffer B; 66-73 min, 21 to 42% of buffer B; 73-82 min, 42 to 95 % of buffer B; 82-90 min, 95% of buffer B). The eluted phosphopeptides were ionized and detected by the TimsTOF pro mass spectrometry. Mass range was 100-1700 m/z. Ion mobility was 0.6-1.6. Collision energy was 20-59 eV. The temperature of drying gas was 180 °C. Capillary voltage was 1.5 KV.

**MS database searching**

MS raw files generated by LC-MS/MS were searched using MaxQuant (version 1.6.17.0.). Protease was Trypsin/P. Up to 2 missed cleavages were allowed. iTRAQ (N-term, K) and carbamidomethyl (C) were considered as fixed modifications. Variable modifications were oxidation (M), acetyl (Protein N-term), and phospho (S/T/Y). The cutoff of false discovery rate (FDR) by using a target-decoy strategy.

**Identification of variant peptides**

MaxQuant search data were analyzed by “R” package (version 2.15) software. Protein, peptide, and phosphorylation-site identifications were filtered, and the screening conditions were as follows: the phosphorylation sites with expression value ≥ 50% in any groups were retained; phosphorylation sites with missing value ≤ 50% were filled with the mean value of the same group, screen localization probability ≥ 0.75, Delta score ≥ 8, and converse data via median normalization and log_2_-transformation to obtain highly reliable phosphorylation sites. Based on obtained highly reliable phosphorylation sites, the differences between control and defactinib (10 μM) groups were calculated, and the MOMO (http://meme-suite.org/tools/momo) software was applied to analyze the motif of phosphorylation sites to obtain the specific substrates. Then, Uniprot, KEGG databases were used to annotate the function of phospho-proteins between control and defactinib (10 μM) groups. The phosphoproteomic data have been deposited in <https://www.iprox.cn/page/home.html>, and the accession number was: PXD032254.

**Untargeted metabolomics**

**Sample preparation**

1 mL of CAFs CM were transferred to a 5 mL Eppendorf tube, and then lyophilized. 400 μL mixture of methanol and water (1/4, vol/vol) were added to each sample. Samples were vortexed for 30 s, sonicated for 3 min, and then added with 10 μL internal standard (2-chloro-L-phenylalanine; 0.3 mg/mL, dissolved in methanol). Mixed samples were placed at -20 °C for 2 h, and then centrifuged at 13, 000 *g* for 10 min at 4 °C. 150 μL supernatants from each sample were collected using crystal syringes (filtered through 0.22 μm microfilters) and transferred to LC vials, which were stored at -80 °C for further LC-MS assay.

**LC-MS analysis**

Metabolic analyses were performed on an UPLC Ultimate 3000 system (Dionex) coupled to a A-Exactive mass spectrometer (ThermoFisher). The chromatographic separation was conducted using ACQUITY UPLC HSS T3 column (100 mm × 2.1 mm, 1.8 μm; Waters) with temperature was set at 45 °C. The mobile phases A and B were consisted of water and acetonitrile both containing 0.1 % formic acid, and run at a flow rate of 0.35 mL/min. The linear gradient was as follows: 0.01 min, 5% of phase B; 2 min, 5% of phase B; 4 min, 30 % of phase B; 8 min, 50 % of phase B; 10 min, 80 % of phase B; 14-15 min, 100 % of phase B; 15.1-18 min, 5 % of phase B. The mass spectrometer was performed in a full-scan mode ranging from 100-1000 m/z, running at a 70, 000 resolution in both positive and negative modes simultaneously to obtain metabolites.

**Untargeted lipidomic**

**Sample preparation**

After lyophilized, lipids from CAFs CM were extracted by 400 μL mixture of chloroform and methanol (2/1, vol/vol) in the presence of 20 μL internal standard (Lyso PC-17:0, 0.1 mg/mL), and then vortexed for 30 s, sonicated for 3 min, plated for 30 min at -20 °C. Subsequently, samples were centrifuged at 12, 000 *g* for 10 min at 4 °C, and volatilized. The residue of lipids was re-dissolved with 200 μL isopropanol and methanol (1/1, vol/vol), vortexed for 30 s, sonicated for 3 min, and then centrifuged at 12, 000 *g* for 10 min at 4 °C. 150 μL supernatants were collected for further study.

**LC-MS analysis**

Lipidomic analyses were conducted using an UPLC Ultimate 3000 system (Dionex) coupled to a A-Exactive mass spectrometer (ThermoFisher). The chromatographic separation was conducted using ACQUITY UPLC BEH C8 column (100 mm × 2.1 mm, 1.7 μm; Waters) with temperature was set at 55 °C. The mobile phase A was consisted of acetonitrile and water (6/4, vol/vol) containing 10 mM ammonium acetate. The mobile phase B was consisted of isopropanol and acetonitrile (9/1, vol/vol) containing 10 mM ammonium acetate. The flow rate was set at 0.26 mL/min. The linear gradient was as follows: 0.00 min, 32 % of phase B; 1.5 min, 32 % of phase B; 15.5 min, 85 % of phase B; 15.6 and 18.0 min, 97 % of phase B; 18.1 and 21.0 min, 32 % of phase B. The mass spectrometer was performed in a full-scan mode ranging from 100-1500 m/z, running at a 70, 000 resolution in both positive and negative modes simultaneously to obtain lipids.

**Pseudo-targeted Lipidomics**

**Sample preparation**

30 cases plasma samples from ESCC patients with 4 cases stage Ⅰ and 26 cases stage Ⅱ and Ⅲ, were subjected to pseudo-targeted lipidomics. The lipid was extracted from 100 μL plasma using 300 μL choroform-methanol (2:1, v/v, supplementing with 0.1 mM BHT) contained 20 μL known amounts of 74 isotope-labeled internal mix standards (identify 1, 000 lipids), vortex for 30 seconds and ultrasonic extraction for 10 minutes, and then hold 30 minutes at -20 °C, centrifuge 10 minutes (13, 000 rpm) at 4 °C, 200 μL chloroform layers were transferred into a centrifuge tube. Residues were reextracted according to above condition. Combine the chloroform layers together and lyophilize in a centrifugal vacuum evaporator at 4 °C. Samples were reconstituted in isopropanol-methanol (1:1, v/v), vortexed for 30 seconds, ultrasonically extracted for 3 minutes, centrifuged 10 minutes (13, 000 rpm) at 4 °C, and then stored at -20 °C. Finally, 150 μL supernatants were subjected to LC/MS analysis.

**LC-MS analysis**

LC-MS analysis was conducted using an ExionLC^TM^ system (ABSCIEX) coupled to Qtrap 6500 plus system (ABSCIEX) equipped with an IonDrive^TM^ Turbo V source. The chromatographic separation was conducted using ACQUITY UPLC BEH C8 column (100 mm × 2.1 mm, 1.7 μm; Waters) with temperature was set at 55 °C. The mobile phase A was consisted of acetonitrile and water (6/4, vol/vol) containing 0.1 % formic acid and 10 mM ammonium formate. The mobile phase B was consisted of isopropanol and acetonitrile (9/1, vol/vol) containing 0.1 % formic acid and 10 mM ammonium formate. The flow rate was set at 0.35 mL/min. The sample injection volume was 5 μL. The linear gradient was as follows: 0.00 min, 0 % of phase B; 1.5 min, 0 % of phase B; 5.0 min, 55 % of phase B; 15.0 min, 90 % of phase B; 16.0 and 18.0 min, 100 % of phase B; 18.1 and 20.0 min, 0 % of phase B. The MS analysis was conducted in the negative/positive-ion mode working in the time-scheduled MRM method to high-throughoutly screen more than 1000 lipids. The source condition is as follow: curtain gas is 35 psi, the ion spray (IS) voltage is -4500 V/+5500 V, source temperature is 350 °C, and Gas 1 and Gas 2 is 40 psi and 45 psi.

**Data analysis**

The acquired data were analyzed using the MRMPROBS software^1^. Relevant parameters were set as follows: smoothing level is 2, minimum peak width is 5, minimum peak height is 500, retention time tolerance is 0.2 min, and supplemented by manual correction. The concentration of lipids in plasma from different tumor stages of ESCC patients (30 cases) were divided into two groups, including Stage Ⅰ and stage Ⅱ /Ⅲ, and the difference of lipid level between these two groups was analyzed using Mann-Whitney U test. Variables are expressed as the median with interquartile range (IQR) in Supplementary Table 1.

**Reference:**

1. Zhao D, Zhang J, Zhang LY, et al. PAFR/Stat3 axis maintains the symbiotic ecosystem between tumor and stroma to facilitate tumor malignancy. *Acta Pharm Sin B.* **13,** 694-708 (2023).

2. Tsugawa H, Kanazawa M, Ogiwara A, et al. MRMPROBS suite for metabolomics using large-scale MRM assays. Bioinformatics. **30,** 2379-2380 (2014).

**Supplementary Figure legends and Figures**


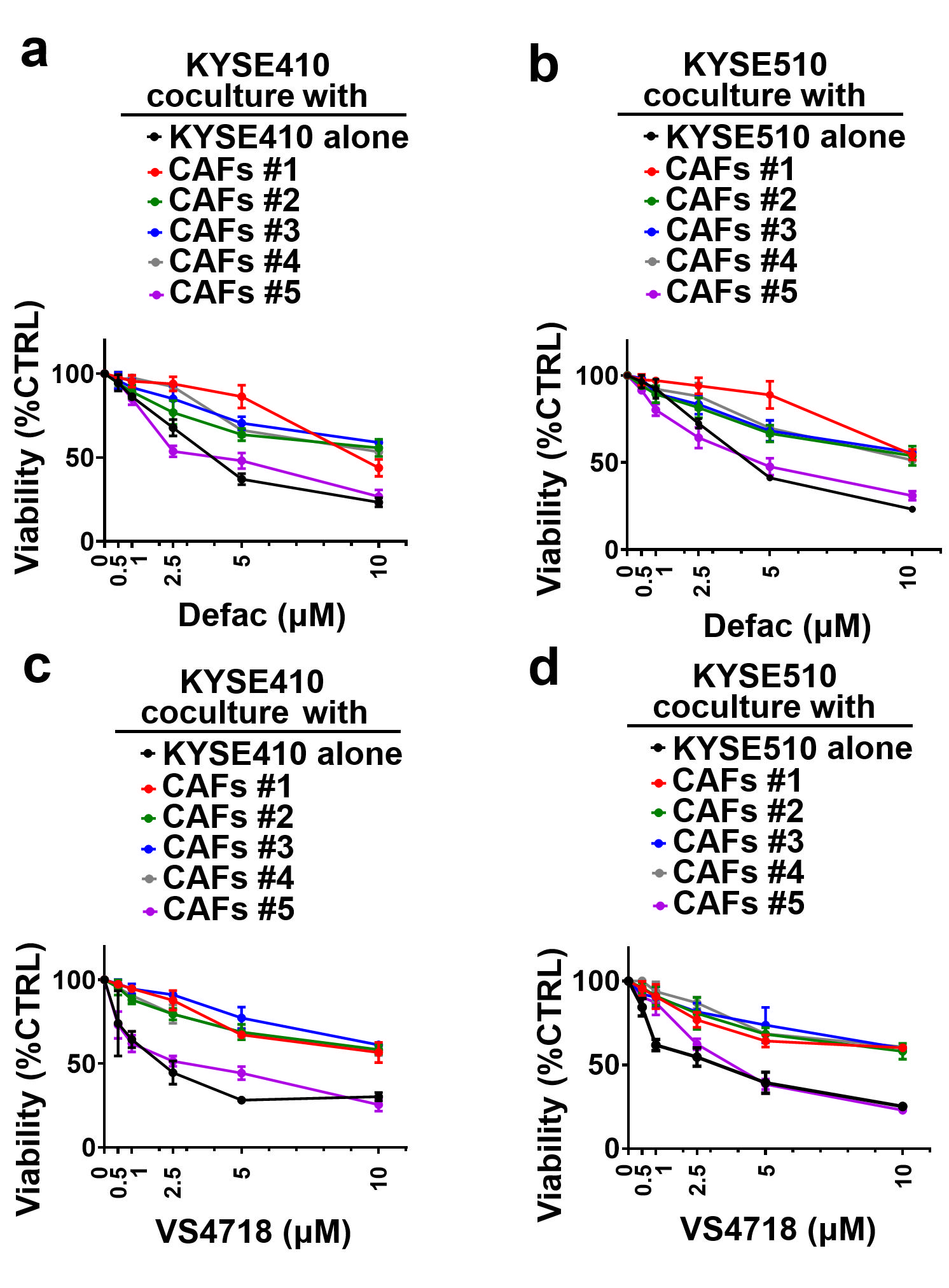


**Supplementary Figure 1. CAFs mediate defactinib and VS4718 resistance in KYSE410 or KYSE510 cells/CAFs coculture system**

(a-d) The dose-response curve of Fig. 1b. (a-b) The dose-response curve of defactinib (0-10 μM) in KYSE410 (a) or KYSE510 (b) cells cultured alone or cocultured with CAFs #1 to #5. (c-d) The dose-response curve of VS4718 (0-10 μM) in KYSE410 (c) or KYSE510 (d) cells cultured alone or cocultured with CAFs #1 to #5.


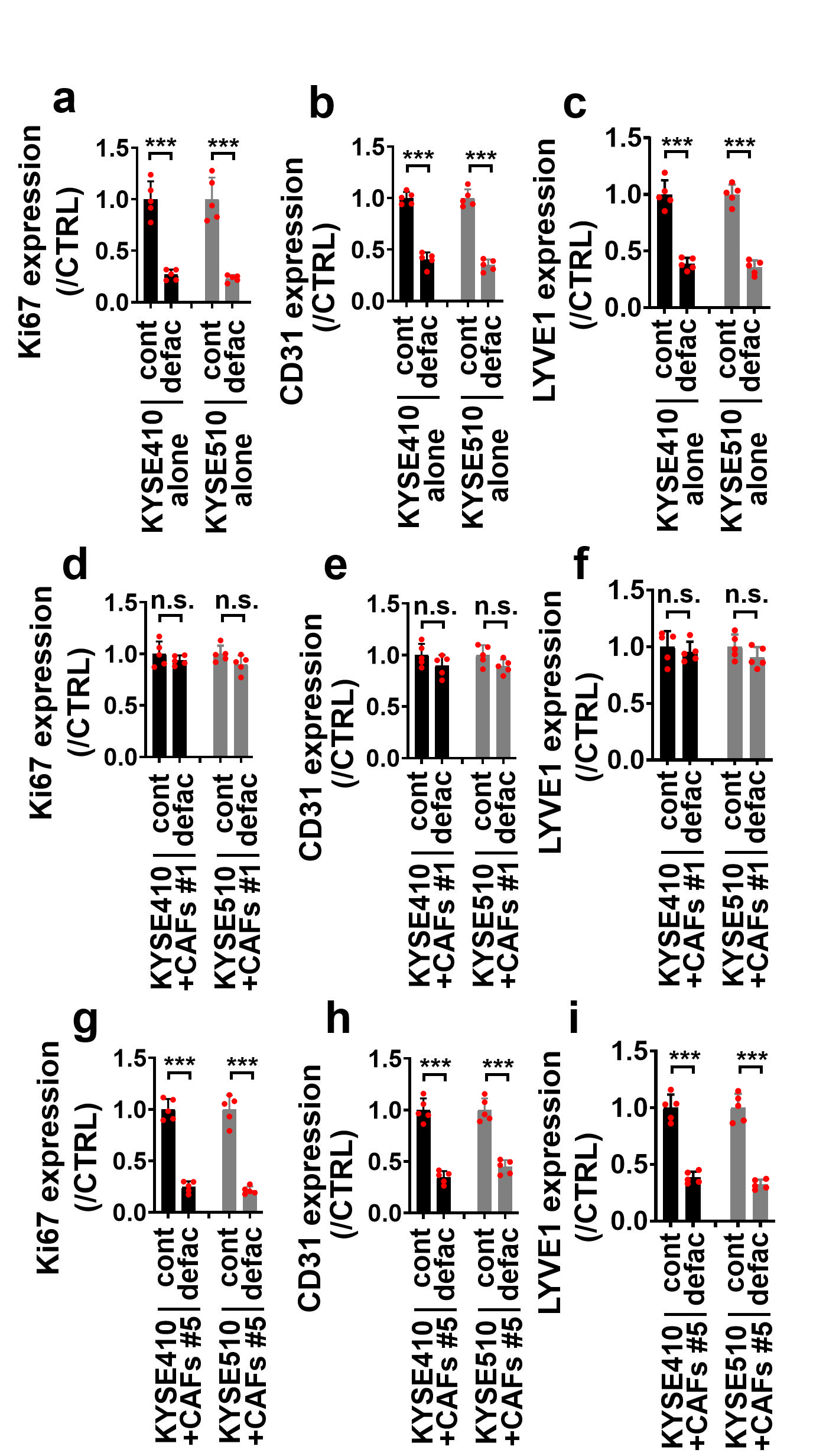


**Supplementary Figure 2. CAFs mediate defactinib resistance in ESCC treatment in xenografted model**

(a-i) The experimental condition of Supplementary Fig. 2 was consistent with that of Fig. 1d and e. After tumors were resected on day 27, the expressions of Ki67 (a, d, g), CD31 (b, e, h), and LYVE1 (c, f, i) in indicated ESCC tumor alone (a, b, c), ESCC/CAFs #1 (d, e, f) or CAFs #5 (g, h, i) coinjection mouse model were assessed using quantitative ELISA assays. n.s. no significant difference; *** *P* < 0.001. Error bars, mean ± SD of five independent experiments.


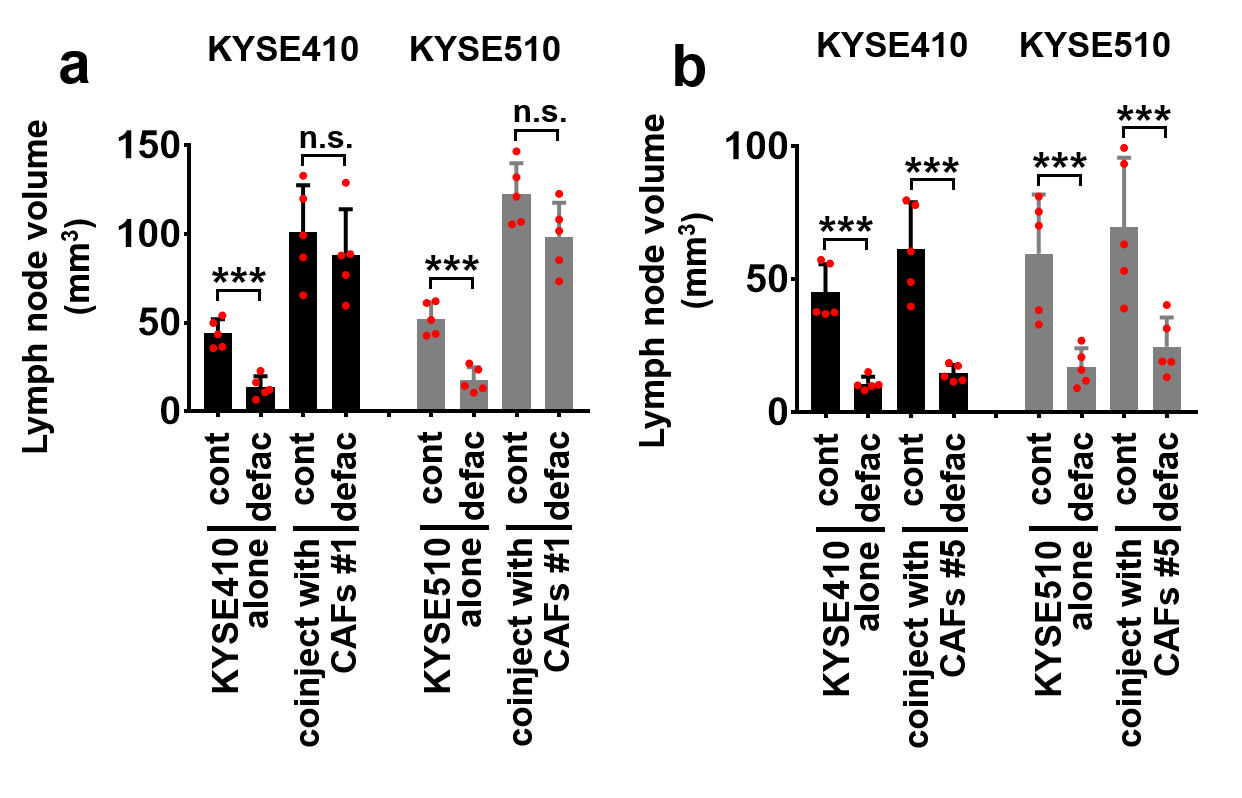


**Supplementary Figure 3.** **CAFs mediate defactinib resistance in the popliteal lymph node metastasis model**

(a-b) A popliteal lymph node metastasis model was established in mice by inoculating the foot pads with KYSE410 or KYSE510 cells and CAFs #1 (a) or #5 (b). After 1 week, mice were treated with control vehicle or defactinib (25 mg/kg/day, p.o.) for 4 weeks. The lymph nodes were enucleated and lymph node volume was calculated. n.s. no significant difference; *** *P* < 0.001. Error bars, mean ± SD of five independent experiments.


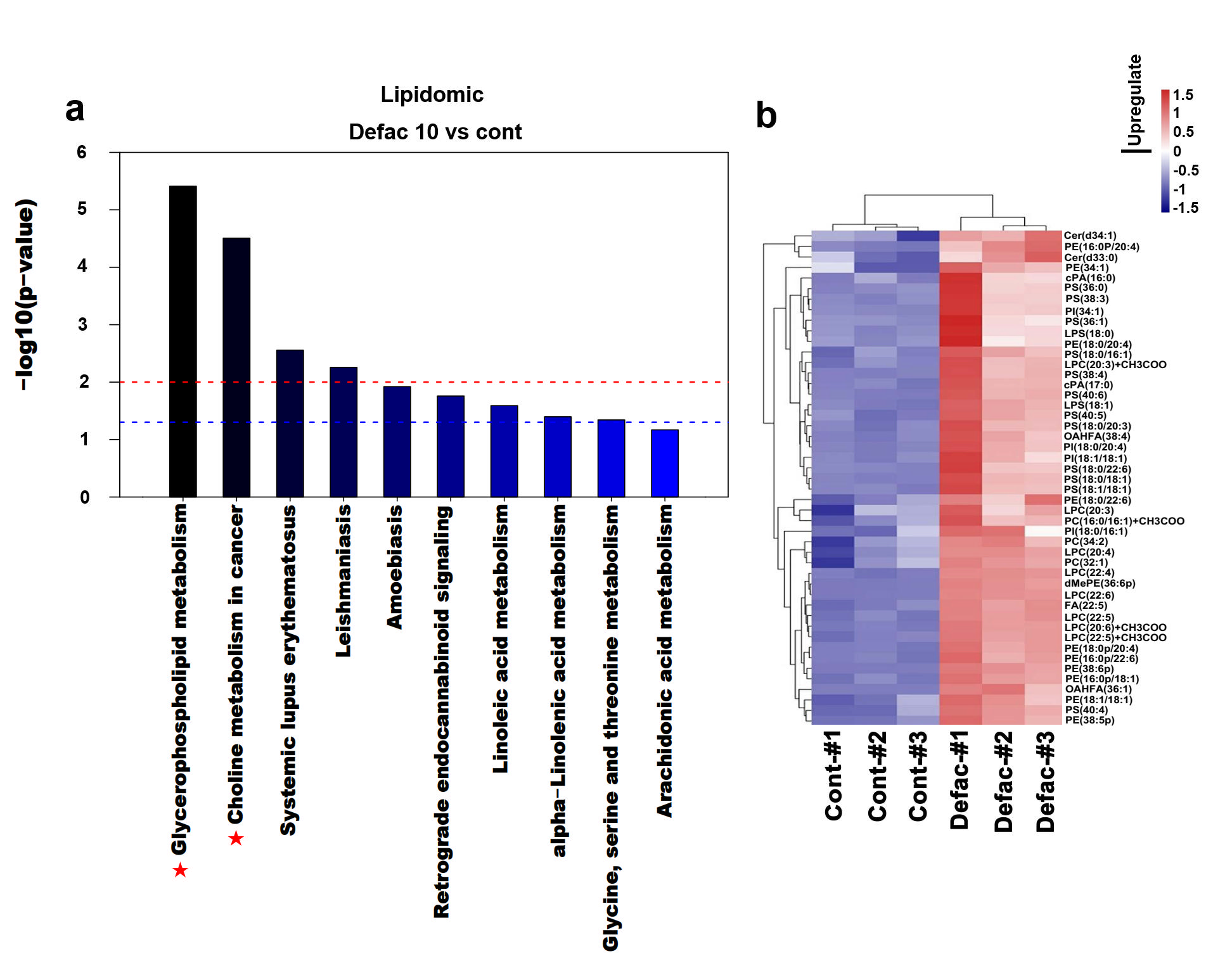


**Supplementary Figure 4. Lipidomics identifies that glycerophospholipid and choline metabolisms were enriched in CAFs treated with defactinib**

(a) CAFs #1 were treated with control or defactinib (10 μM) for 24 hours, and then CM were collected, and subjected to untargeted lipidomics. The 10 enriched pathways have been shown using bar chart. (b) The defactinib (10 μM) upregulated lipid metabolites were shown using heatmap.


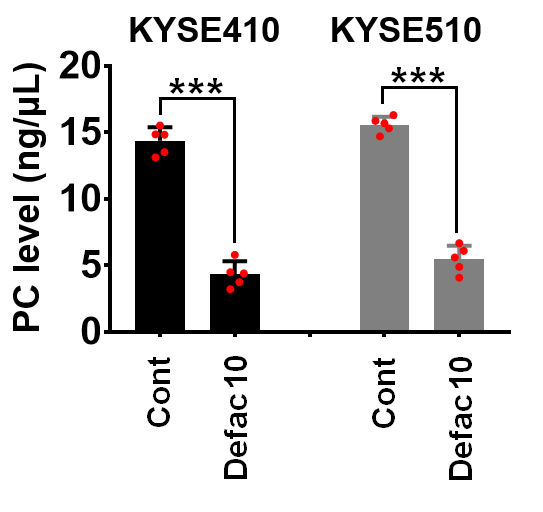


**Supplementary Figure 5. Defactinib inhibits the secretion of PCs from ESCC cells**

Levels of PCs secreted from KYSE410 and KYSE510 cells with/without 10 μM defactinib, were evaluated using quantitative PCs ELISA assay.


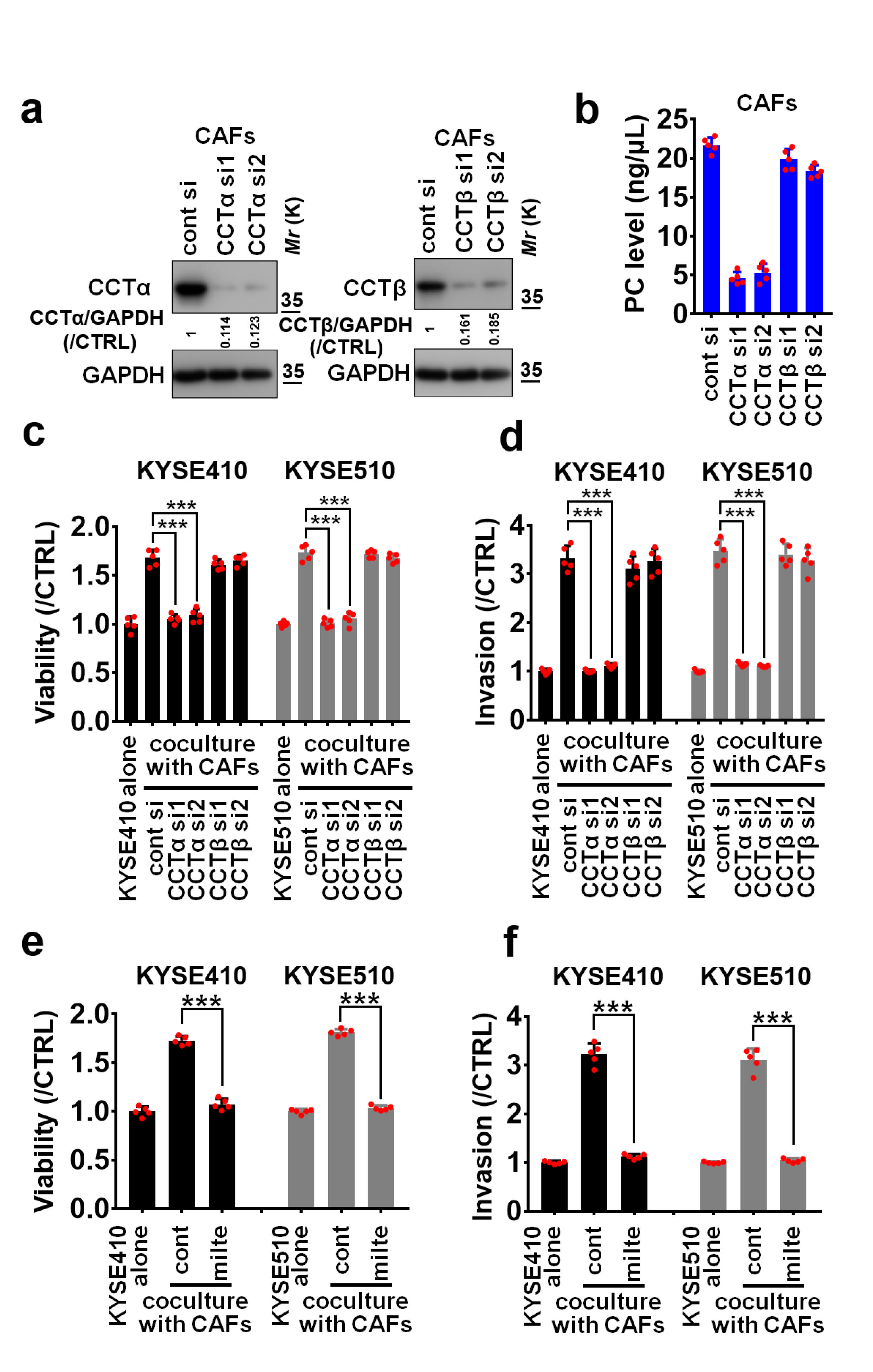


**Supplementary Figure 6. CAFs-derived AKT2/CCTα axis promotes ESCC malignancy**

(a) CAFs #1 were transfected with control siRNA, CCTα siRNA1/2, or CCTβ siRNA1/2. The transfected efficacy of siRNAs was evaluated using immunoblotting assay. GAPDH was used as the loading control. (b) The secreted PCs from indicated CAFs #1 were measured using quantitative PCs ELISA assay. (c) Transwell apparatus with 0.4 μm pore size was used to evaluate the CAFs #1-derived CCTα or CCTβ-mediated growth of tumor cells. The control siRNA, CCTα siRNA1/2, or CCTβ siRNA1/2 CAFs #1 were plated in the upper chamber of transwell plates. KYSE410 or KYSE510 cells were respectively plated in the lower chamber of transwell plates, and cocultured with indicated CAFs #1 for 4 days, and then growth of indicated ESCC cells was measured using MTS assay. (d) Indicated CAFs #1 were cultured in Transwell apparatus with 8 μm pore size, KYSE410 or KYSE510 cells were cultured in the upper chamber of transwell plates for 24 hours. The invasion of KYSE410 or KYSE510 cells was evaluated using Transwell invasion assay. (e) CAFs #1 were plated in the upper chamber of transwell plates with 0.4 μm pore size. KYSE410 or KYSE510 cells were respectively plated in the lower chamber of transwell plates, and cocultured with CAFs #1 with/without miltefosine (25 μM) for 4 days, and then growth of indicated ESCC cells was measured using MTS assay. (f) CAFs #1 were cultured in Transwell apparatus with 8 μm pore size, KYSE410 or KYSE510 cells were cultured in the upper chamber of transwell plates and treated with/without miltefosine (25 μM) for 24 hours. The invasion of KYSE410 or KYSE510 cells was evaluated using Transwell invasion assay. *** *P* < 0.001. Error bars, mean ± SD of five independent experiments.


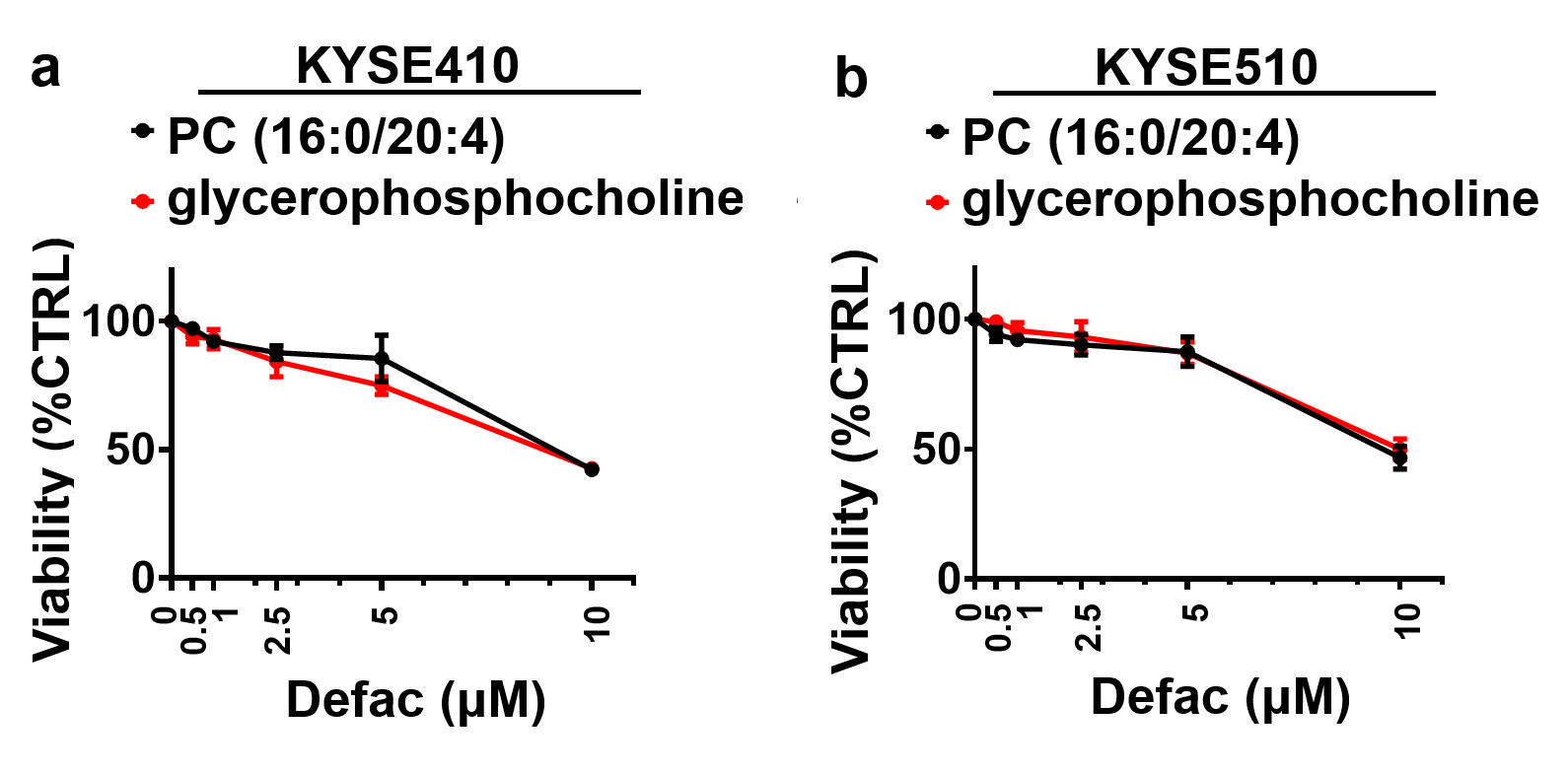


**Supplementary Figure 7. PCs mediated the resistance of defactinib in ESCC treatment**

(a-b) The dose-response curve of Fig. 3a. The dose-response curve of defactinib (0-10 μM) in KYSE410 (a) or KYSE510 (b) cells cultured with 10 μM PC (16:0/20:4) or glycerophospholipid with defactinib (0-10 μM) for 4 days, then growth of indicated ESCC cells was evaluated using MTS assay.


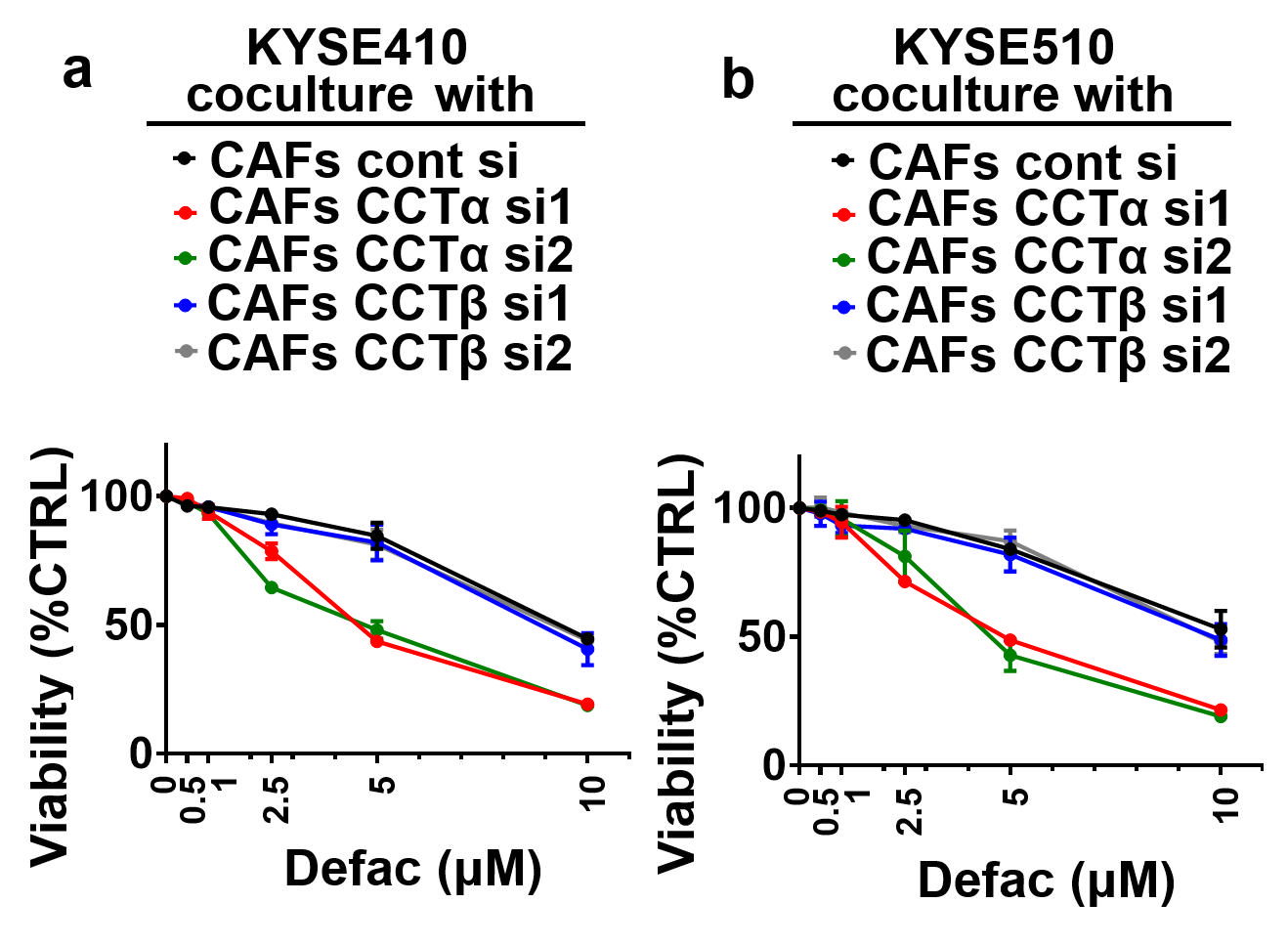


**Supplementary Figure 8. CAFs-derived CCTα mediated the resistance of defactinib in ESCC treatment**

(a-b) The dose-response curve of Fig. 3b. The dose-response curve of defactinib (0-10 μM) in KYSE410 (a) or KYSE510 (b) cells cocultured with CAFs #1 harbored control siRNA, CCTα siRNA1, 2, or CCTβ siRNA1, 2 for 4 days, then growth of indicated ESCC cells was evaluated using MTS assay.


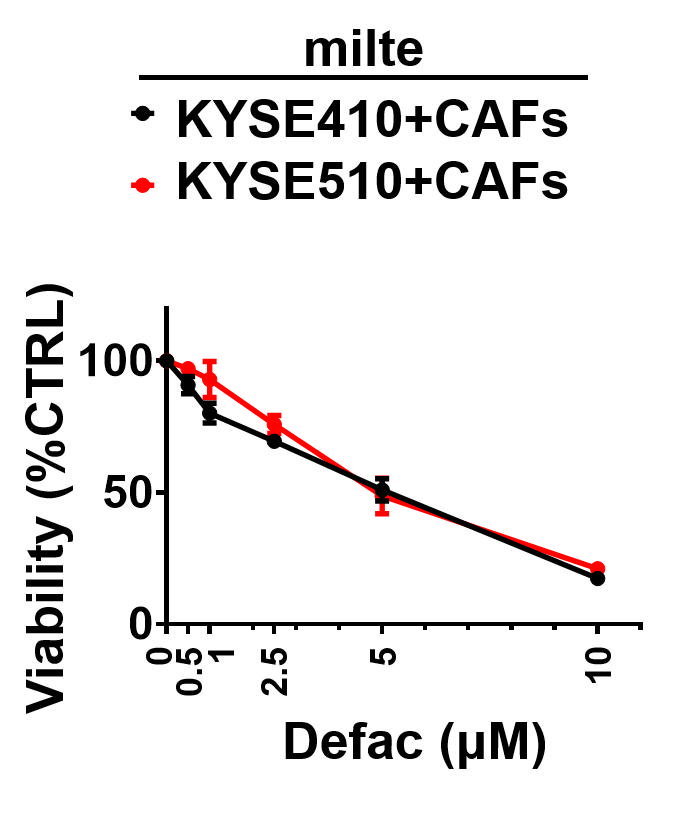


**Supplementary Figure 9. Miltefosine enhanced the tumor inhibitory effect of defactinib in ESCC/CAFs coculture system**

The dose-response curve of Fig. 3c. The dose-response curve of defactinib (0-10 μM) combined with miltefosine (25 μM) in KYSE410 or KYSE510 cells cocultured with CAFs #1 for 4 days, then growth of indicated ESCC cells was evaluated using MTS assay.


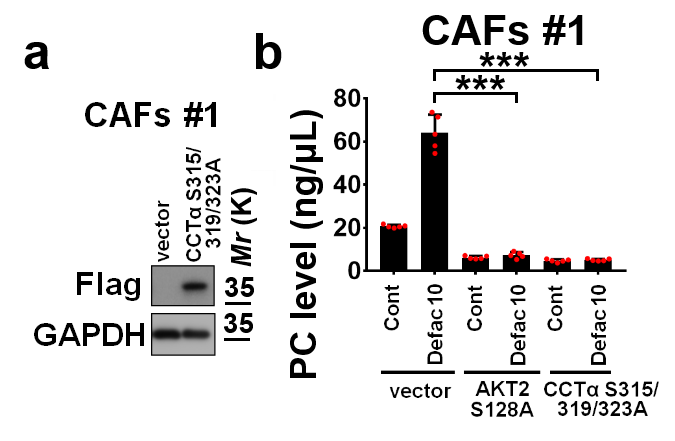


**Supplementary Figure 10. Function loss of AKT2/CCTα axis in CAFs impaired defactinib-stimulated PCs secretion from CAFs**

(a) CAFs #1 were stably transfected with control vector, loss-of-function CCTα S315/319/323A plasmid, and the transfection efficacy was evaluated using immunoblotting to detect the expression of Flag. GAPDH was used as the loading control. (b) CAFs #1 harbored control vector, AKT2 S128A, or CCTα S315/319/323A mutant were treated with/without defactinib (10 μM) for 24 hours, and the secreted PCs was evaluated using quantitative PCs ELISA assay. *** *P* < 0.001. Error bars, mean ± SD of five independent experiments.


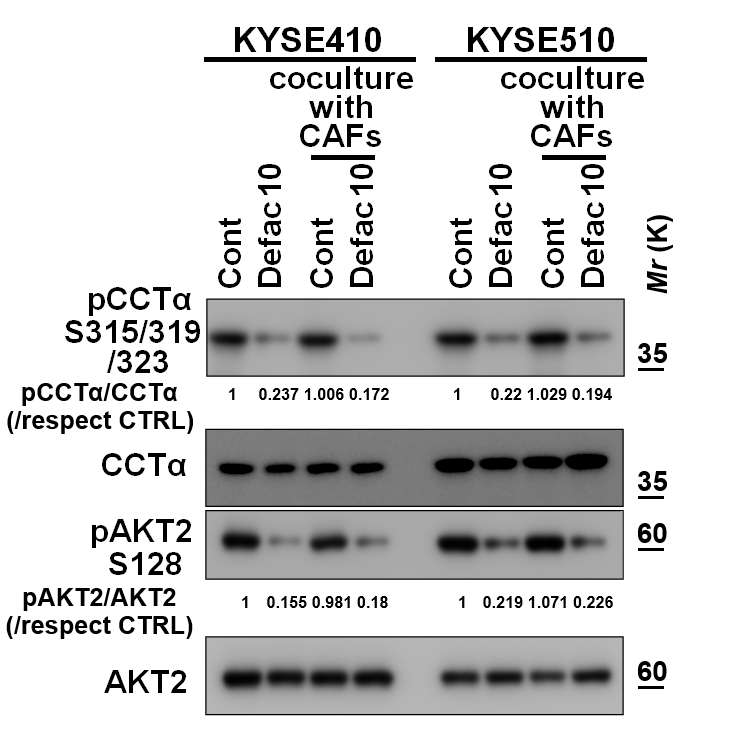


**Supplementary Figure 11. Defactinib inhibits the activation of AKT2/CCTα axis in ESCC cells**

KYSE410 or KYSE510 cells (cultured in the low chamber of transwell plates with 0.4 μm pore size) in the presence or absence of CAFs #1 (cultured in the upper chamber of transwell plates with 0.4 μm pore size), and treated with 10 μM defactinib for 24 hours. Then, the lysates of KYSE410 or KYSE510 cells were collected, and the expression of pCCTα Ser^315/319/323^, CCTα, pAKT2 Ser^128^, or AKT2 was evaluated using immunoblotting assay.


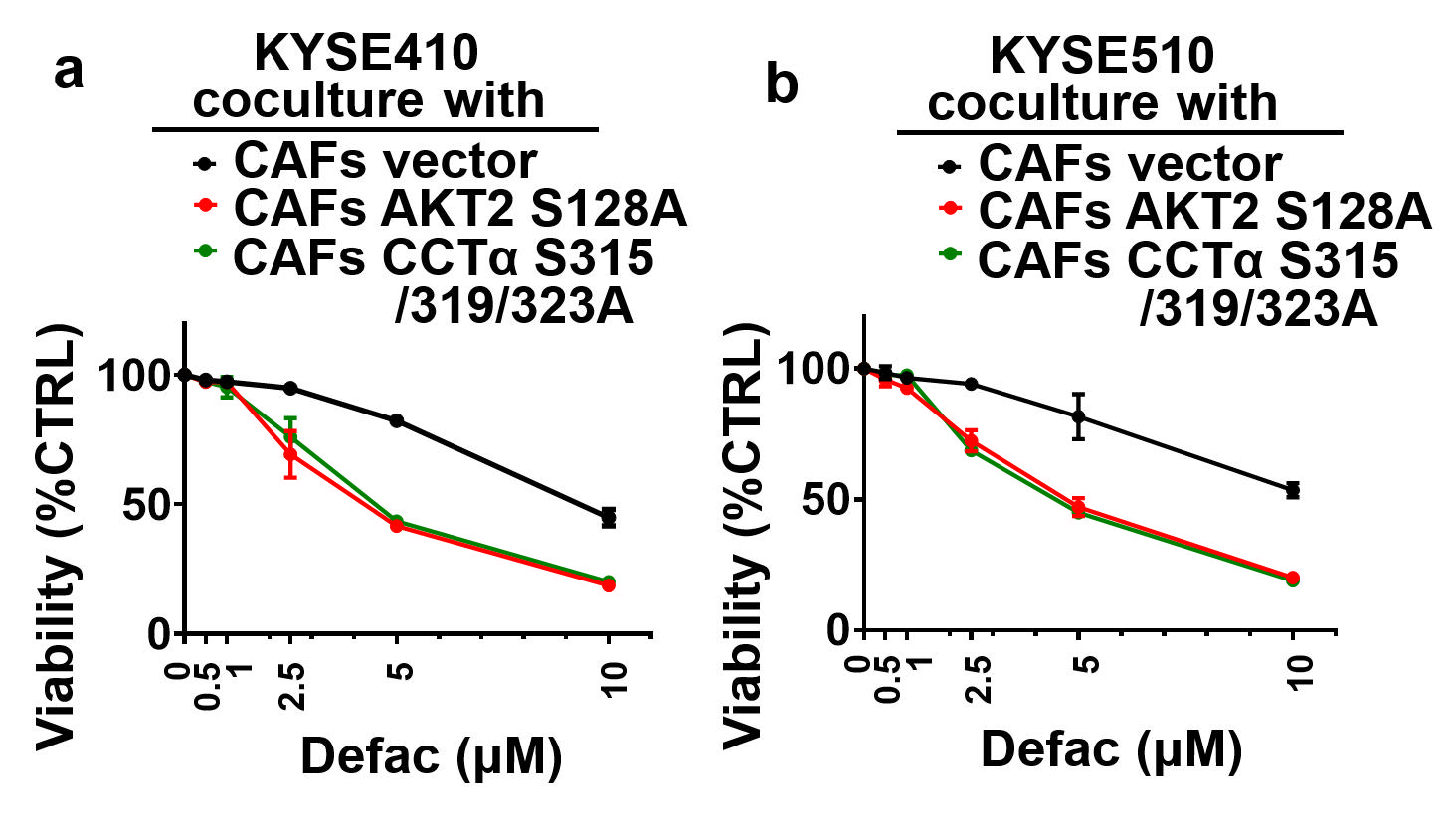


**Supplementary Figure 12. Function loss of AKT2/CCTα axis in CAFs enhanced defactinib-mediated growth inhibitory effect on ESCC cells**

(a-b) The dose-response curve of Fig. 5a. The dose-response curve of defactinib (0-10 μM) in KYSE410 (a) or KYSE510 (b) cells cocultured with CAFs #1 harbored vector, loss-of-function AKT2 (S128A) or CCTα (S315/319/323A) mutant for 4 days, then growth of indicated ESCC cells was evaluated using MTS assay.


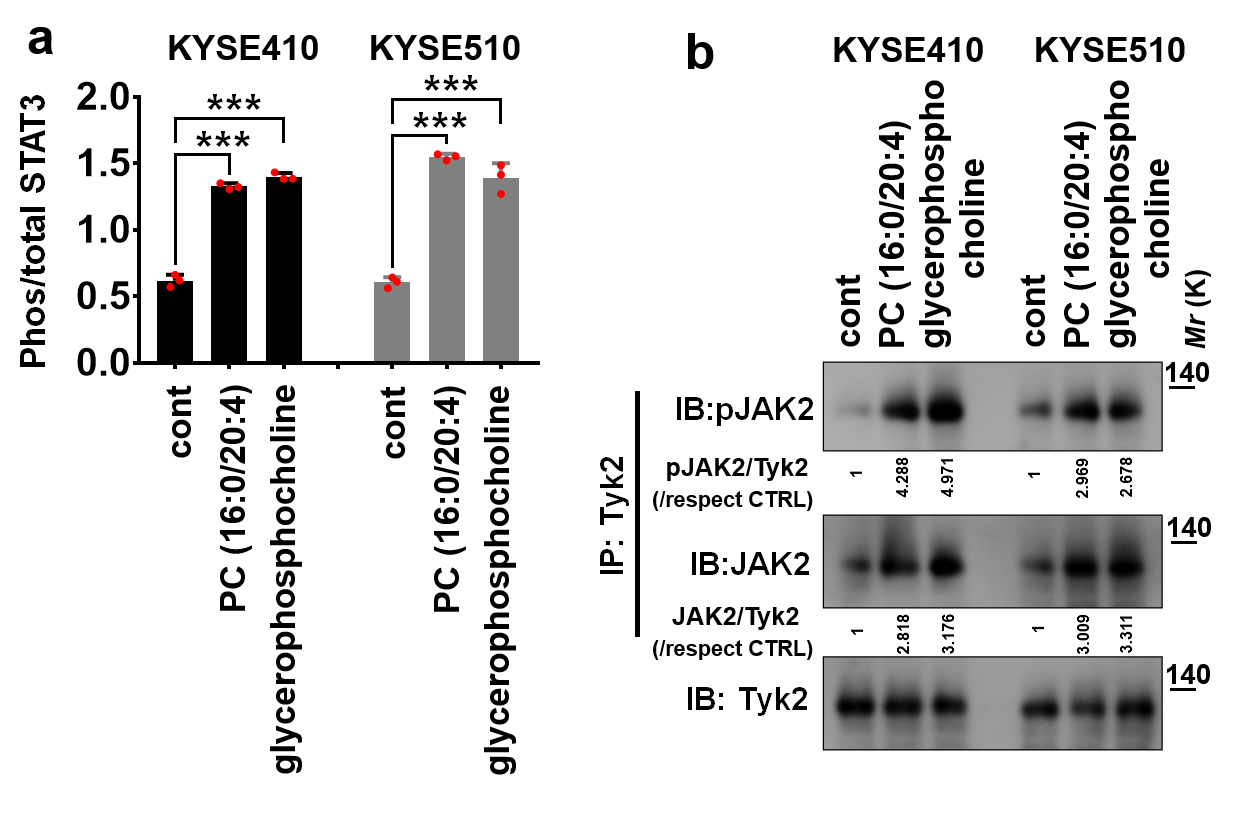


**Supplementary Figure 13. PCs stimulate the activation of intratumoral JAKs/STAT3 pathway**

(a) KYSE410 or KTSE510 cells were respectively treated with 10 μM PC (16:0/20:4) or glycerophospholipid for 24 hours. Then, lysates of KYSE410 and KYSE510 cells were collected, and the STAT3 activity was evaluated using quantitative ELISA assay. (b) The experimental condition of (b) was similar with that of (a). Then, lysates were immunoprecipitated with Tyk2 antibody and subjected to immunoblotting with Tyk2, JAk2, or pJAK2.


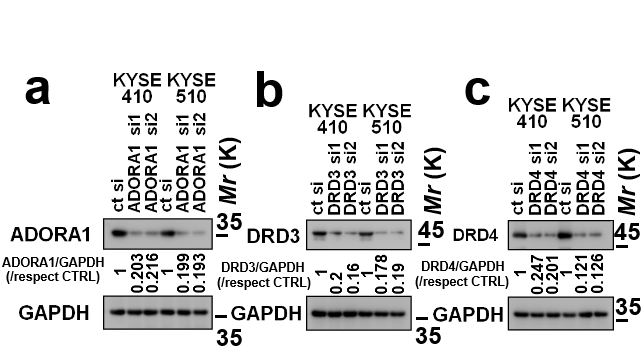


**Supplementary Figure 14. The depletion efficacy of indicated siRNAs in ESCC cells**

(a-c) KYSE410 or KYSE510 cells were respectively transfected with control siRNA, ADORA1 siRNA1/2 (a), DRD3 siRNA1/2 (b), or DRD4 siRNA1/2 (c). The transfected efficacy of siRNAs was evaluated using immunoblotting assay. GAPDH was used as the loading control.


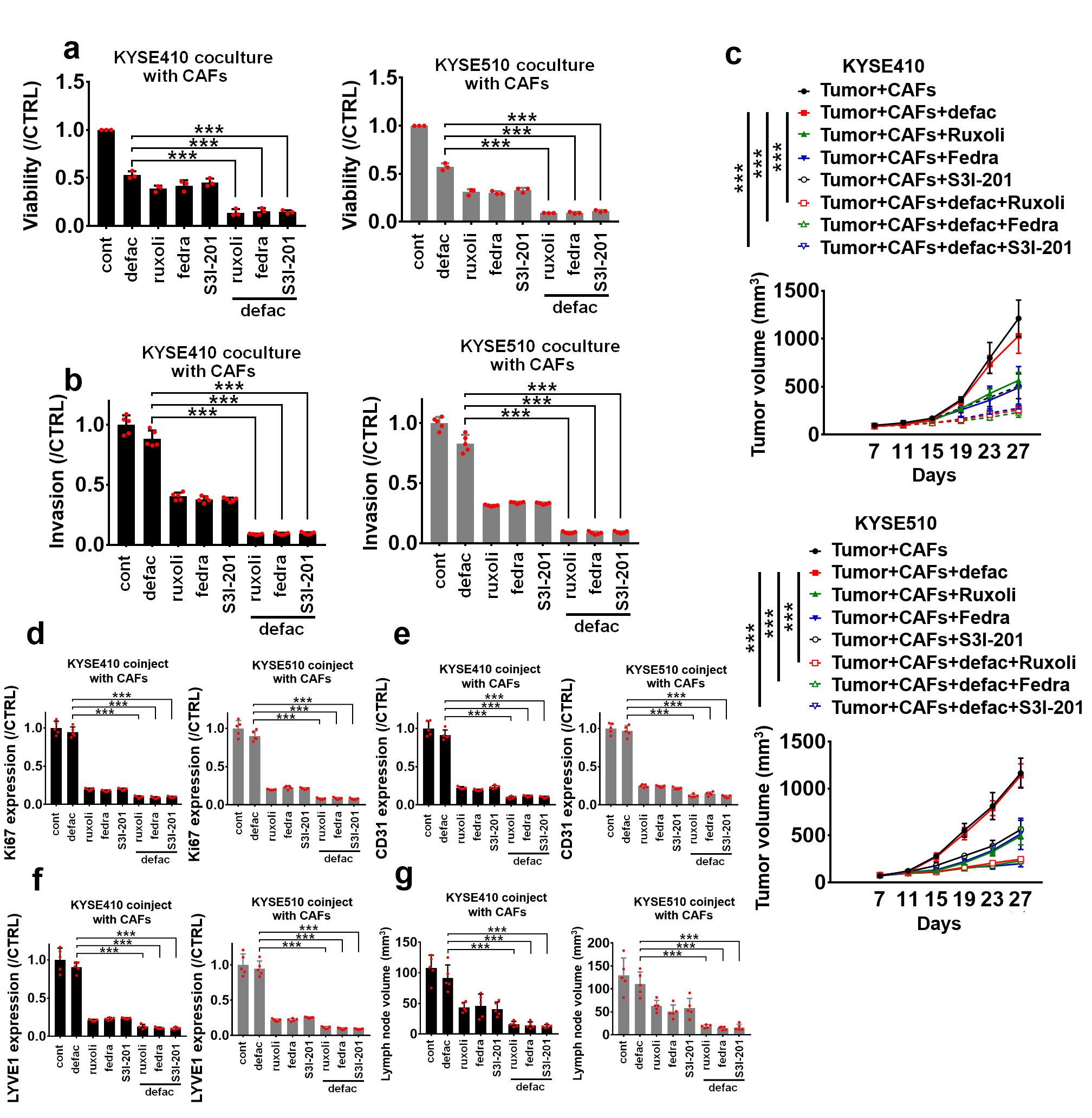


**Supplementary Figure 15. Synergistic inhibition of FAK and JAK2/STAT3 pathways blocks CAFs-mediated ESCC malignancy**

(a) Transwell apparatus with 0.4 μm pore size was used to evaluate the tumor growth inhibitory effect of defactinib and JAK2 inhibitors-ruxolitinib, or fedratinib, or STAT3 inhibitor-S3I-201. CAFs #1 were plated in the upper chamber of transwell plates. KYSE410 (left panel) or KYSE510 (right panel) cells were respectively plated in the lower chamber of transwell plates. After cells were seeded, defactinib (10 μM), ruxolitinib (10 μM), or fedratinib (10 μM), or S3I-201 (20 μM) alone or defactinib (10 μM) in combination with ruxolitinib (10 μM), or fedratinib (10 μM), or S3I-201 (20 μM), were added, incubated for 4 days, and then growth of indicated ESCC cells was measured using MTS assay. (b) Transwell apparatus with 8 μm pore size was used to evaluate the anti-invasive ability of defactinib and inhibitors of JAK2/STAT3 pathways, CAFs #1 were plated in the lower chamber of transwell plates. KYSE410 (left panel) or KYSE510 (right panel) cells were respectively plated in the upper chamber of transwell plates. After cells were seeded, inhibitors similar with (a) were incubated for 24 hours, and then tumor invasion was measured using transwell invasion assay. (c) KYSE410 (upper panel) or KYSE510 (lower panel) cells were respectively coinjected with CAFs #1 into the flank of BALB/c mouse. After the xenografts reached at approximately 80-100 mm^3^. Tumor cells with CAFs #1 were treated with control vehicle or defactinib (25 mg/kg/day, p.o.), ruxolitinib (10 mg/kg/day, p.o.), or fedratinib (10 mg/kg/day, p.o.), or S3I-201 (25 mg/kg/day, p.o.) alone or defactinib (25 mg/kg/day, p.o.) in combination with ruxolitinib (10 mg/kg/day, p.o.), or fedratinib (10 mg/kg/day, p.o.), or S3I-201 (25 mg/kg/day, p.o.). Tumor volume was measured every 4 days for the indicated period. Curves of tumor volume were listed. (d-f) After tumors were resected on day 27, the expression of Ki67 (d), CD31 (e) and LYVE1 (f) was in KYSE410 (left panel) or KYSE510 (right panel) tumors assessed using quantitative ELISA assays. (g) A popliteal lymph node metastasis model was established in mice by inoculating the foot pads with KYSE410 (left panel) or KYSE510 (right panel) cells and CAFs #1. After 1 week, mice were treated with inhibitors of FAK or/and JAK2/STAT3 pathways (all treatment conditions and doses of indicated agents were according to Supplementary Fig. 15c for 4 weeks. The lymph nodes were enucleated and lymph node volume was calculated. *** *P* < 0.001. Error bars, mean ± SD of three to five independent experiments.


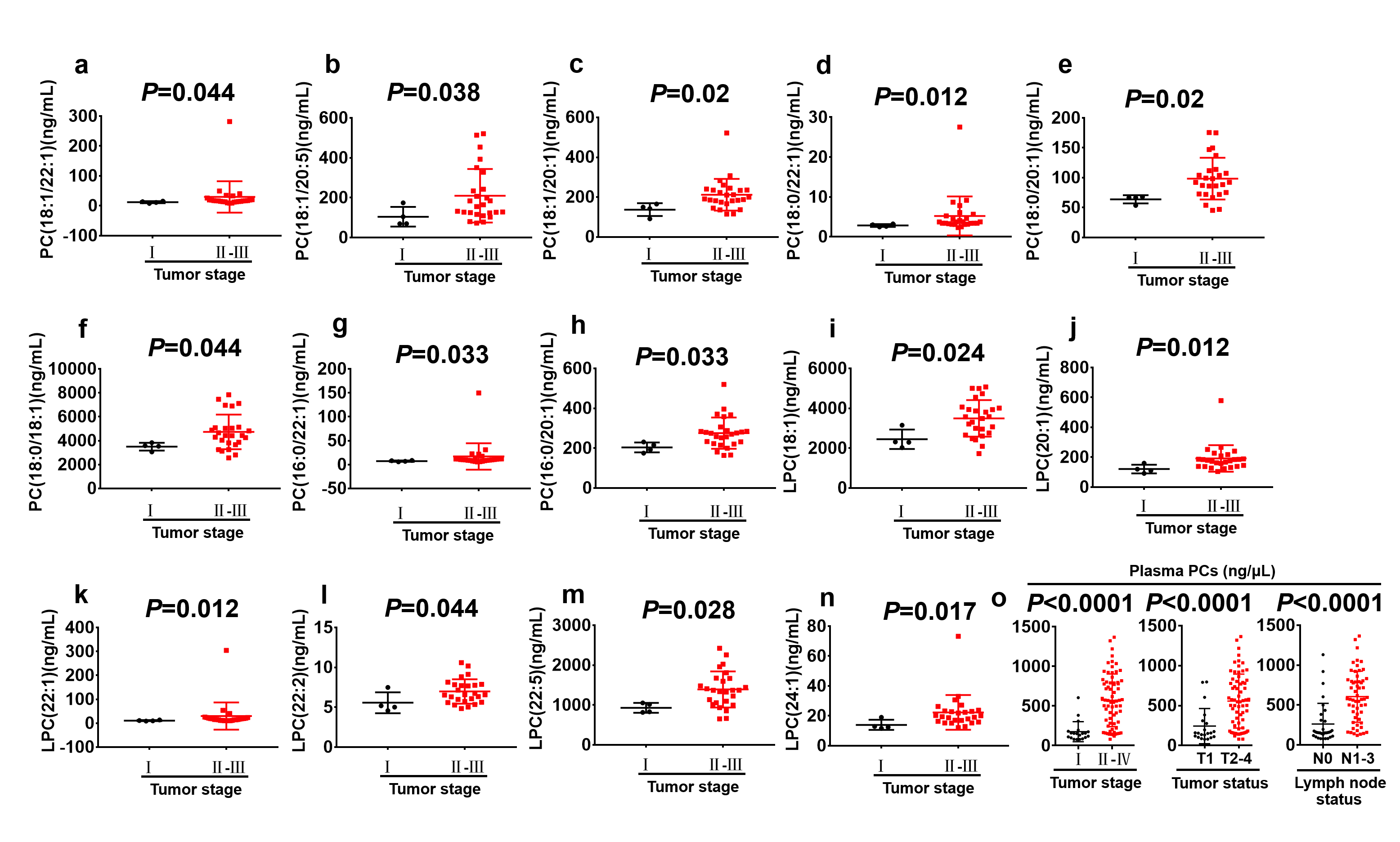


**Supplementary Figure 16. Plasma PCs serve as biomarkers for ESCC diagnosis**

(a-n) Pseudo-targeted lipidomics was applied to quantitatively and comprehensively screen the 1, 000 lipids in plasma from 30 cases ESCC patients with 4 cases stage Ⅰ and 26 cases stage Ⅱ and Ⅲ. Several PCs and their metabolites-LPCs, including PC (18:1/22:1) (a), PC (18:1/20:5) (b), PC (18:1/20:1) (c), PC (18:0/22:1) (d), PC (18:0/20:1) (e), PC (18:0/18:1) (f), PC (16:0/22:1) (g), PC (16:0/20:1) (h), LPC (18:1) (i), LPC (20:1) (j), LPC (22:1) (k), LPC (22:2) (l), LPC (22:5) (m), or LPC (24:1) (n), were statistically higher in stage Ⅱ and Ⅲ than in stage Ⅰ. (o) PCs assay was used to evaluate the levels of PCs in plasma of 89 cases ESCC patients (stage Ⅰ: 20 cases, stage Ⅱ-Ⅳ: 69 cases; T1: 21 cases, T2-4: 68 cases; N0: 32 cases, N1-4: 57 cases). Mann-Whitney U test. *P* value was shown.


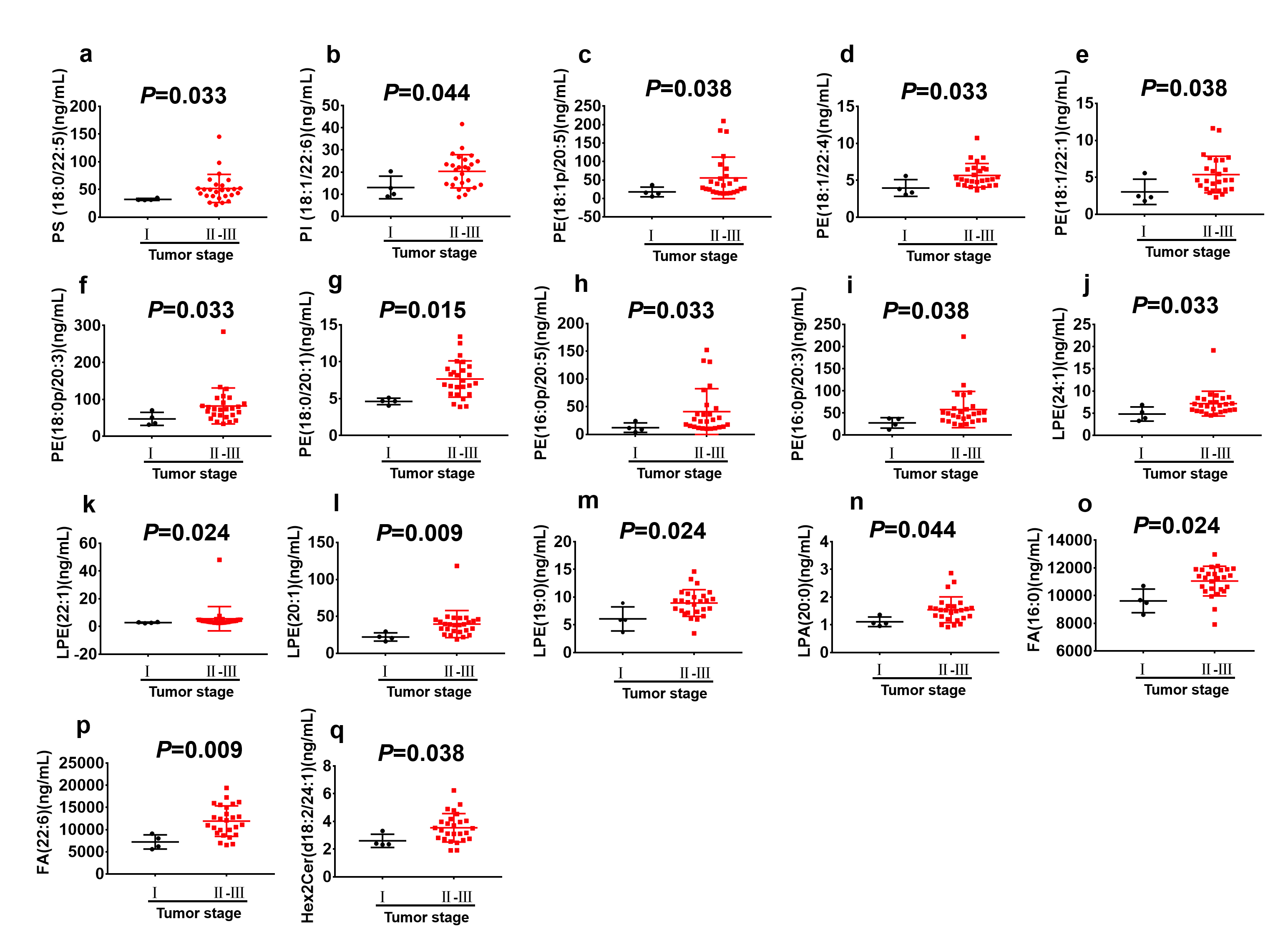


**Supplementary Figure 17. Plasma lipids serve as biomarkers for ESCC diagnosis**

(a-q) Pseudo-targeted lipidomics was applied to quantitatively and comprehensively screen the 1, 000 lipids in plasma from 30 cases ESCC patients with with 4 cases stage Ⅰ and 26 cases stage Ⅱ and Ⅲ. Several lipids, including PS (18:0/22:5) (a), PI (18:1/22:6) (b), PE (18:1p/20:5) (c), PE (18:1/22:4) (d), PE (18:1/22:1) (e), PE (18:0p/20:3) (f), PE (18:0/20:1) (g), PE (16:0p/20:5) (h), PE (16:0p/20:3) (i), LPE (24:1) (j), LPE (22:1) (k), LPE (20:1) (l), LPE (19:0) (m), LPA (20:0) (n), FA (16:0) (o), FA (22:6) (p), or Hex2Cer (d18:2/24:1) (q), were statistically higher in stage Ⅱ and Ⅲ than in stage Ⅰ. Mann-Whitney U test. *P* value was shown.


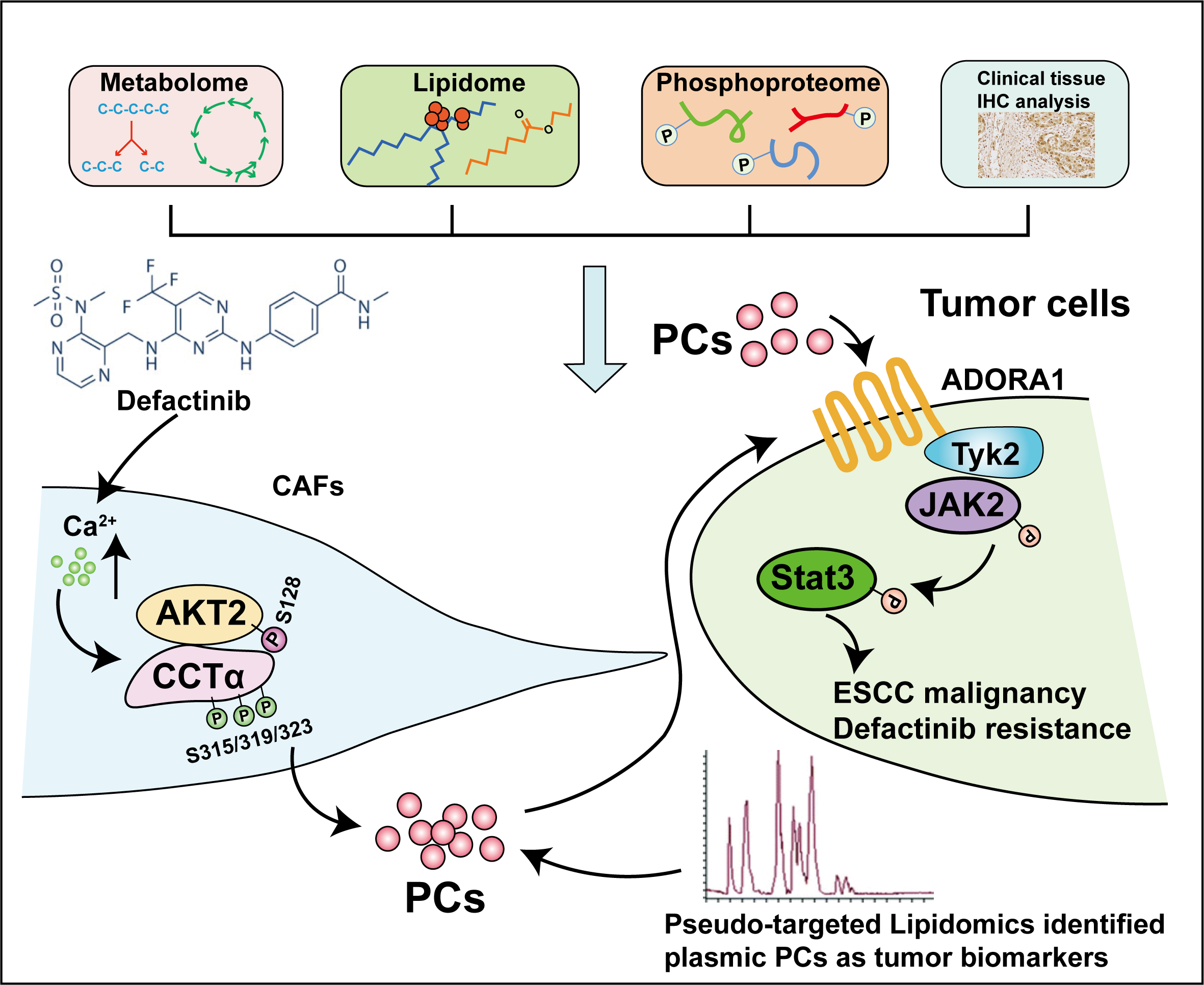


**Supplementary Figure 18. Proposed model of regulation of PCs secretion from CAFs by defactinib**

Defactinib activates stromal AKT2/CCTα axis to phosphorylate Ser^315/319/323^ sites in CCTα, which facilitates the secretion of PCs from CAFs, and then stimulates the activity of intratumoral JAK2/STAT3 pathway to mediated defactinib resistance. Plasma PCs serves as biomarkers for evaluation of ESCC malignancy. Present figure is drawn using Adobe illustrator 2022 software (<https://www.adobe.com/products/illustrator/>).
