# Supplementary material for: AKT2S128/CCTαS315/319/323-positive cancer-associated fibroblasts (CAFs) mediate focal adhesion kinase (FAK) inhibitors resistance via secreting phosphatidylcholines (PCs)
Source: Signal Transduct Target Ther. 2024 Jan 28;9:21. doi: 10.1038/s41392-023-01728-6 (PMC10821909; doi:10.1038/s41392-023-01728-6)
Supplement: Supplementary file 3 — Supplementary Tables 2 and 3 [file 41392_2023_1728_MOESM3_ESM.doc]

**Supplementary Table 2: antibodies and reagents**

| **REAGENT or RESOURCE** | **SOURCE** | **Cat Number** |
| --- | --- | --- |
| **Antibodies** |  |  |
| GAPDH | Cell Signaling Technology | 5174S |
| Phospho-FAK (Tyr397) Monoclonal Antibody | Invitrogen | 44-625G |
| AKT2 (L79B2) Mouse mAb | Cell Signaling Technology | 5239S |
| Phospho-AKT2 (Ser128) | Synthesized by ABclonal |  |
| CCTα (D18B6) Rabbit mAb | Cell Signaling Technology | 6931S |
| Phospho-CCTα (Ser315/319/323) | Synthesized by ABclonal |  |
| Anti-CCTβ Antibody | Abcam | ab127200 |
| Phospho-STAT3(Tyr705) | Cell Signaling Technology | 9145 |
| Anti-alpha smooth muscle Actin antibody | Abcam | ab5694 |
| Tyk2 | Cell Signaling Technology | 14193 |
| JAK2 | Cell Signaling Technology | 3230 |
| pJAK2 (Tyr1007/1008) | Cell Signaling Technology | 3776 |
| ADORA1 | Abclonal | A5219 |
| ADORA2A | Abclonal | A1587 |
| ADORA2B | Abclonal | A1953 |
| DRD1 | Abclonal | A2893 |
| DRD2 | Abclonal | A12930 |
| DRD3 | Abclonal | A4587 |
| DRD4 | Abclonal | A1337 |
| CHRM1 | Abclonal | A16819 |
| Muscarinic ACM2 | Abclonal | A5154 |
| CHRM3 | Abclonal | A1602 |
| CHRM4 | Abclonal | A2866 |
| HTR1A | Abclonal | A2801 |
| HTR1B | Abclonal | A18285 |
| HTR2A | Abclonal | A20538 |
| HTR2B | Abclonal | A5670 |
| ADRA1A | Abclonal | A9410 |
| ADRA1B | Abclonal | A7872 |
| ADRA2A | Abclonal | A2809 |
| ADRA2B | Abclonal | A8525 |
| ADRA2C | Abclonal | A16956 |
| ADRB1 | Abclonal | A20818 |
| ADRB2 | Abclonal | A2048 |
| **Chemicals** |  |  |
| Defactinib (FAK inhibitor) | Selleck | S7654 |
| VS4718 | Selleck | S7653 |
| Glycerophosphocholine | Cayman Chemical | 20736 |
| PC (16:0/20:4) | Cayman Chemical | 25658 |
| Miltefosine (CCT inhibitor) | MCE | HY-13685 |
| BAPTA-AM | Selleck | S7534 |
| **Commercial Kits** |  |  |
| Phosphatidylcholine Assay Kit | Sigma-Aldrich | MAK049 |
| Phospho-STAT3 (Tyr705) and Total STAT3 ELISA kit | RayBiotech | PEL-Stat3-Y705-T |
| Human Ki-67/MKI67 ELISA Kit | RayBiotech | ELH-MKI67-1 |
| Human PECAM-1 (CD31) ELISA Kit | RayBiotech | ELH-PECAM1-1 |
| Human LYVE-1 ELISA Kit | RayBiotech | ELH-LYVE1-1 |
| Cell Invasion Assay | biovision | K913 |
| Calcium Assay Kit | Nanjing Jiancheng | C004-3-1 |

**Supplementary Table 3: Sequences of siRNAs**

| **Oligonucleotides** |  |
| --- | --- |
| **Product Name** | **Sequence** |
| siPCYT1A (CCTα)_1 | GCTTCACGGTGATGAACGA |
| siPCYT1A (CCTα)_2 | GAGAGTTTATGCCGATGGA |
| siPCYT1B (CCTβ)_1 | GAGCCATGATCTAATTCAA |
| siPCYT1B (CCTβ)_2 | GAGCCCTTATGCAAGCAAA |
| siADORA1_1 | ACCTCCCATTGACGAGGAT |
| siADORA1_2 | CCGTGATCAAGTGCGAGTT |
| siDRD3_1 | GTACAGCCAGCATCCTTAA |
| siDRD3_2 | GTCCTTGTCTATGCCAGAA |
| siDRD4_1 | GCCGAGTTCCGCAACGTCT |
| siDRD4_2 | GGACGCCCTTCTTCGTGGT |
